# Response surface optimization of a cardioprotective compound through pharmacosomal drug delivery system: in vivo bioavailability and cardioprotective activity potential

**DOI:** 10.1007/s13346-023-01315-w

**Published:** 2023-04-05

**Authors:** Marwa H. S. Dawoud, Mai A. Zaafan, Sarah S. Saleh, Islam M. Mannaa, Nabila M. Sweed

**Affiliations:** 1 Department of Pharmaceutics, Faculty of Pharmacy, October University for Modern Sciences and Arts, 6th of October City, Egypt; 2 Department of Pharmacology and Toxicology, Faculty of Pharmacy, October University for Modern Sciences and Arts, 6th of October City, Egypt; 3 Department of Analytical Chemistry, Faculty of Pharmacy, October University for Modern Sciences and Arts, 6th of October City, Egypt

**Keywords:** Cardioprotective activity, Central composite design, Vanillic acid, Bioavailability, Optimization, Release rate

## Abstract

Vanillic acid (VA) is a phenolic compound with potential antioxidant activity, which improves ischemia-induced myocardial degeneration, by reducing oxidative stress; however, it suffers poor bioavailability owing to its poor solubility. VA-loaded pharmacosomes were optimized using a central composite design, where the effect of phosphatidylcholine:VA molar ratio and the precursor concentration were studied. An optimized formulation (O_1_) was prepared and tested for the release rate of VA, in vivo bioavailability, and cardioprotective potential on myocardial infarction-induced rats. The optimized formulation showed a particle size of 229.7 nm, polydispersity index of 0.29, and zeta potential of − 30 mV. O_1_ showed a sustained drug release for 48 h. The HPLC–UV method was developed for the determination of VA in plasma samples using protein precipitation. The optimized formulation showed a great improvement in the bioavailability as compared to VA. The residence time of the optimized formula was 3 times longer than VA. The optimized formulation showed a more potent cardioprotective effect as compared to VA, via inhibition of the MAPK pathway with subsequent inhibition of PI3k/NF-κB signaling, in addition to its antioxidant effect. The optimized formulation showed normalization of many oxidative stress and inflammatory biomarkers. Thus, a VA-loaded pharmacosome formulation with promising bioavailability and cardioprotective activity potential was prepared.

**Graphical Abstract:**

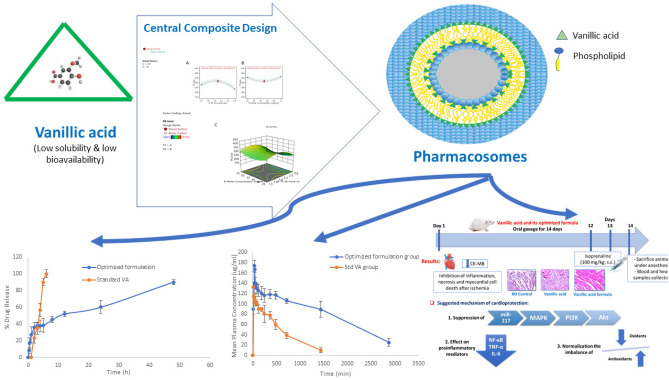

## Introduction

Recently, natural compounds and natural drug products have gained great attention in the treatment of various diseases and disorders, where different substances with a variety in their applications have been tailored for several therapies [1]. Vanillic acid (VA), a dihydroxy benzoic acid derivative is one of these natural compounds, obtained from *Angelica sinensis*, and has been widely used in traditional Chinese medicine [2]. VA is present in several fruits as well such as olives, and cereal grains (e.g., whole wheat), in wine, beer, and cider [3].

VA is an intermediate compound that is obtained from the conversion of ferulic acid to vanillin. Moreover, it is also produced when caffeic acid is metabolized [4]. It has been widely used as an antioxidant, antiapoptotic, immunostimulant, and hepatoprotective, and to treat some neurological disorders such as Alzheimer’s disease and Parkinson’s disease [2]. Furthermore, VA was reported to have a promising cardioprotective effect, owing to its ability to decrease oxidative stress and improve myocardial dysfunction [5, 6].

However, VA has poor solubility which leads to its poor bioavailability, of about 25% [7], as it is well known for its dissolution rate-limited bioavailability [8]. Thus, the formulation of VA into nanocarriers could help in the enhancement of its bioavailability.

Pharmacosomes are drug delivery systems in which the drug is covalently complexed with the phospholipid [9]. Phospholipid complexation and its formulation to pharmacosomes are used in many drug formulations especially for drugs with poor solubility and bioavailability [10]. The amphiphilic nature of the phospholipids allows them to modify the drug solubility as well as its release profile. This, in turn, enhances the drug absorption across the biological membranes, and consequently its bioavailability [11]. It has been proven that these drug delivery systems have extreme stability and are capable of improving drug solubility and minimizing the gastrointestinal toxicity of drugs. These systems also show facilitated membrane, tissue, or cell wall transfer, within the organism [12]. Furthermore, the formation of the drug-lipid complex, which is accomplished by a covalent bond, could control the release of the drug from these systems and enhance its stability by reducing the drug’s leaching from these systems, unlike many other lipid formulations [9]. Thus, pharmacosomes could be a promising drug delivery system for VA to overcome its low solubility.

Hence, the aim in the current study is to formulate and optimize VA-loaded pharmacosomes, to enhance VA’s solubility and bioavailability, and to investigate its potential use as a cardioprotective agent.

## Materials and methods

### Materials

Ethanol, dichloromethane, hydrochloric acid, and hematoxylin and eosin (H&E) were purchased from Merck (Darmstadt, Germany). Lecithin, 90% soybean, was obtained from Alfa Aesar (Erlenbachweg 1 Kandel, Germany). Tetrahydrofuran was purchased from Nabhi chemicals (Maharashtra, India). N-Octanol was procured from HeBei GuanLang Biotechnology Co., Ltd. (Shanghai, China), and disodium hydrogen phosphate from Oxford Lab Chem (Maharashtra, India). Isoprenaline hydrochloride, syringic acid (internal standard: IS) of purity ≥ 95%, methanol (HPLC grade), acetic acid, and formic acid (analytical grade) were purchased from Sigma-Aldrich (MA, USA). Vanillic acid was kindly provided by Mahitab H. Elbishbishy from the Department of Pharmacognosy, Faculty of Pharmacy, October University for Modern Sciences and Arts.

## Animals

All animal experiments were conducted in accordance with ARRIVE guidelines, after approval of MSA’s ethical committee (#PT12/EC12/2022). For the in vivo study, male Wistar albino rats weighing 150–200 g were used. They were bought from EGYVAC (Cairo, Egypt) and given free access to water and pellet chow. Rats were housed in plastic cages in the animal house of MSA University under constant conditions (temperature 25 °C, humidity 50%).

## Methods

### Preparation of vanillic acid-loaded pharmacosomes

Pharmacosomes were prepared by the refluxing method, followed by thin film hydration. First, vanillic acid-phosphatidylcholine complex (VA-PC) was prepared, by dissolving VA and phosphatidylcholine (PC) with different molar ratios, in tetrahydrofuran, in a 100-mL round-bottom flask, as shown in Table 1. This solution was refluxed at 60 °C for 3 h to form the complex. Tetrahydrofuran was then evaporated under vacuum at 60 °C, using a rotary evaporator (Heidolph 2, Schwabach, Germany), till a thin film was formed on the wall of the flask. The thin film was then placed in a desiccator for 12 h, to get rid of any residual solvent [13, 14].

**Table 1:** Factor levels and their responses, together with the formulations and their composition, from the central composite design

**Factors**	**Levels of factors**				
	**−α**	**−1**	**0**	** + 1**	** + α**
X_1_–PC: VA molar ratio	0.989	1.300	2.050	2.800	3.110
X_2_ – Precursor concentration (mg/mL)	14.644	25.000	50.000	75.000	85.355

**Responses**	**Desirability Constraints**
Y_1_– Particle size (nm) (PS)	Minimize
Y_2_– Polydispersity index (PDI)	Minimize
Y_3_– Zeta potential (mV) (ZP)	Maximize

**Formulation code**	**Factor level**		**Responses**		
	X_1_	X_2_ (mg/mL)	Y_1_ (nm)	Y_2_	Y_3_ (mV)
F_1_	2.050	50.000	313.4 ± 9.67	0.44 ± 0.02	−31.9 ± 0.98
F_2_	2.800	25.000	270.6 ± 5.76	0.48 ± 0.03	−33.8 ± 1.09
F_3_	1.300	25.000	304.2 ± 4.61	0.37 ± 0.05	−19.0 ± 0.89
F_4_	2.050	50.000	324.8 ± 5.09	0.39 ± 0.08	−32.7 ± 0.73
F_5_	1.050	85.355	416.3 ± 2.67	0.66 ± 0.01	−28.6 ± 1.10
F_6_	2.800	75.000	276.2 ± 3.67	0.48 ± 0.03	−37.2 ± 0.84
F_7_	2.050	50.000	322.4 ± 8.6 0	0.41 ± 0.09	−33.1 ± 0.99
F_8_	3.110	50.000	168.5 ± 4.69	0.37 ± 0.01	−39.2 ± 0.63
F_9_	0.989	50.000	241.2 ± 3.80	0.25 ± 0.03	−21.6 ± 0.63
F_10_	2.050	14.644	406.3 ± 6.29	0.53 ± 0.03	−19.6 ± 0.94
F_11_	1.300	75.000	311.2 ± 3.57	0.37 ± 0.07	−22.6 ± 0.83
F_12_	2.050	50.000	315.7 ± 2.54	0.45 ± 0.04	−32.4 ± 0.62
F_13_	2.050	50.000	306.7 ± 1.76	0.40 ± 0.06	−30.9 ± 0.98

Pharmacosomal vesicles were produced by hydration of the thin film, using 10 mL of phosphate buffer saline (PBS) at pH 6.8. The film was collected by rotation in a rotary evaporator at 120 rpm for 2 h at 40 °C. The dispersion was stored at 4 °C till further characterization [15].

## Drug content determination

To determine the drug content, 10 µL of the prepared formulation was dissolved in 20 mL of methanol by agitation and sonication. The samples were then filtered through a 0.22-µm syringe filter and measured spectrophotometrically (UV-1700, Shimadzu, Japan) at the predetermined wavelength, *λ*_max_ = 290 nm [16].

## Determination of VA-PC interaction

- Infrared spectroscopy

Infrared spectroscopy was tested on each VA, PC, physical mixture of VA and PC, and VA-PC complex, to estimate the interaction between PC and VA, using an IR spectrophotometer (Shimadzu, Japan), in the wave number region from 3500 to 1000 cm^−1^ [17]. IR spectra were determined using KBr pellets, at 25 °C with an IR source, in the transmission mode.

- Powder X-ray diffraction

The crystal habit modification of VA molecules in the complex form was evaluated by XRD. Dry powder samples of VA, PC, physical mixture, and VA-PC complex were tested using XRD (Bruker AXS D8 Advance Diffractometer). The X-ray generator was operated at 40-kV tube voltages and 30 mA of tube current, using the Cu Kα lines as the source of radiation. The diffraction pattern scanning angle ranged from 4 to 80° of 2*θ* in step scan mode (step width 0.02°/min) [18, 19]

- Differential scanning calorimetry

The thermal behavior of each of VA, PC, physical mixture of both, and the VA-PC complex was studied using DSC, by heating to 2.0 ± 0.2 mg of each individual sample in a covered sample pan under nitrogen gas flow. The investigations were carried out over the temperature range of 35–250 °C with a heating rate of 10 °C/min [16].

## Response surface optimization

Central composite design (CCD) was the response surface design used to study the relationship between different factors, and their interactions, and how they affect the responses, and for the optimization of VA pharmacosomal formulation.

The studied factors were the phosphatidylcholine to drug molar ratio (PC:VA) (*X*_1_) and the precursor concentration (concentration of pharmacosomal constituents) (*X*_2_). The effects of these factors on the particle size (PS), polydispersity index (PDI), and zeta potential (ZP) were tested, each at 5 levels. This resulted in the preparation of 13 formulations, as suggested by the Design-Expert 13.0.5.0^®^ software (Stat-Ease Inc., Minneapolis, USA). The data is presented in Table 1. This resulted in 4 factorial points, 4 axial points, and 5 central points. The model was further evaluated in terms of the coefficient of regression and adequate precision. Statistical analysis was performed using analysis of variance (ANOVA), to detect the significant models and the non-significant lack of fit [20]. The optimization technique was based on numerical desirability to obtain an optimized formulation (*O*_1_) with the mentioned desired constraints, as shown in Table 1.

### Characterization tests

#### Particle size, polydispersity index, and zeta potential measurement

It should be noted that the PS was measured in terms of the hydrodynamic diameter of the particles [21]. Particle size (PS), polydispersity index (PDI), and zeta potential (ZP) were measured by dynamic light scattering method (DLS), using a zetasizer at 25 °C after suitable dilution (Malven Zetasizer version 6.20 serial number: MAL 104 4595, Worcestershire, UK). Samples were measured in triplicate at least for each of the aforementioned measurements [20].

#### Morphological determination of the optimized formulation

The morphology of the prepared pharmacosomes was examined using transmission electron microscopy (TEM), and scanning electron microscopy, as follows.

For TEM examination, a drop of the sample was added on a coated carbon copper grid, forming a thin film. The film was stained with uranyl acetate and lead citrate. The sample was morphologically examined using TEM (JEM-1400 JEOL, Tokyo, Japan) after being dried for contrast enhancement [22].

For scanning electron microscopy examination, samples were coated with gold in a fine coat ion sputter (JEOL JFC-1100, Jyväskylä, Finland). The coated sample was analyzed using a scanning electron microscope (JOEL, JSM-6360LA, Japan), where the results were photographed [23].

#### Saturated solubility and partition coefficient studies

The saturated solubility of VA and VA-loaded pharmacosomes were measured in distilled water. A known excess amount of VA or VA-loaded pharmacosomes was added to 5 mL of distilled water, and stirred for 24 h, in a thermostatically controlled mechanical shaker at 25 °C (incubator shaker, SK Scientific Solutions, Coimbatore, Tamil Nadu). Excess VA was removed by centrifugation at 2292 × g for 5 min, filtered through a 0.22 µm syringe filter, and measured spectrophotometrically at the predetermined wavelength; *λ*_max_ = 286 nm [16].

For the determination of partition coefficient (*P*), the solubility of VA and VA-loaded pharmacosomes were measured in n-octanol, as previously mentioned [16, 17]. The following equation was used for the calculation of *P*:

1 $$P={C}_{o}/{C}_{w}$$

where *C*_o_ was the concentration of the drug in n-octanol, and *C*_w_ was the concentration of the drug in distilled water [24].

#### In vitro drug release

In vitro drug release of standard VA (VA in water) was compared with the optimized formulation (O_1_), using the dialysis membrane method. The dialysis membrane (Spectrum Medical Inc., Los Angeles, CA, USA, molecular weight cut off 12,000–14,000 Da), was completely washed with boiling water prior to use, and was soaked in the release medium overnight, which was then dialyzed for 3 h at 37 °C with gentle shaking of the buffer. Ten mg of standard VA, or O_1_ equivalent to 10 mg VA (vanillic formulation), were placed in the dialysis membrane, which was firmly clipped from both ends to prevent any spillage. The dialysis bag was immersed in 100 mL of phosphate buffer saline (PBS) at pH 6.8, as simulated intestinal conditions, which was placed in a thermostatically controlled mechanical shaker at 37 ± 0.10 °C at 50 rpm for 48 h. Two-milliliter samples were drawn at 0, 0.25, 0.5, 1, 2, 4, 5, 8, 12, 24, and 48 h, and were substituted with freshly prepared buffer to attain sink conditions. The drawn samples were measured spectrophotometrically [22]. The experiment was done in triplicate.

#### Stability study

The optimized formulation (*O*_1_) was tested for stability at 4 °C, for a 3-month period. The sample was stored in tightly closed glass vials. The sample was analyzed for drug content, PS, PDI, and ZP. Moreover, it was visually inspected for any color change or appearance of aggregates [17].

#### Pharmacokinetic study

- Chromatographic system and conditions

The chromatographic system used was Waters 2690 Alliance HPLC system (Waters, UK) using Kinetex C18 (4.6 mm ID × 100 mm L, particle size 5 µm, Phenomenex, USA). The system was equipped with a binary solvent delivery pump, an autosampler, and a photodiode array detector. An isocratic elution was applied using a mobile phase consisting of 20% methanol and 80% acetic acid (0.1%) at a flow rate of 0.8 mL/min. UV detection was set at 272 nm, and the injection volume was 10 µL at ambient temperature.

- Standard solutions and quality control samples

Working standard solutions of VA and internal standard (IS) syringic acid were prepared in methanol, with a concentration of 500 μg/mL. The plasma samples for the calibration curve were prepared in the range of (1–200 µg/mL) for VA spiked with (10 µg/ml) of IS. Three levels of quality control samples were prepared in blank plasma: low (LQC), medium (MQC), and high (HQC) with concentrations of 3, 80, and 160 µg/mL, respectively. The calibration curve of each drug was constructed by plotting the relative peak area against the corresponding concentration, from which the regression equations were calculated.

- Sample preparation

A volume of 400 μL of rat plasma was spiked with 100 μL from the working standard solutions of the IS. A volume of 500 μL of extracting solvent (1% formic acid in methanol) was immediately added to the mixture to precipitate the proteins followed by vigorous vortexing for 2 min. The mixture was centrifuged at 9168 × g for 10 min. Into a Wassermann tube, the clear supernatant was carefully transferred and heated to 60 °C to be concentrated, then reconstituted with the mobile phase. Subsequently10 μL of the supernatant was injected into the HPLC system.

- Method validation

Method validation was carried out according to the FDA Guidance for Industry and Bioanalytical Method Validation [25] with respect to selectivity, linearity, range, LOQ, accuracy, precision, and stability. Extraction recovery and matrix effect were calculated at each QC level using Eqs. 2 and 3:

2 $$\begin{aligned}\mathrm{Extraction\ recovery}\ \%=&\; \text{pre-extracted samples}/\text{post}\\&\text{-extracted samples}\end{aligned}$$

3 $$\text{Matrix effect}= \text{post-extracted samples}/\text{post-neat samples}$$

- Pharmacokinetic study
  Eighteen rats weighing approximately 150–200 g were used and divided randomly into 3 groups (*n* = 6). The first group did not receive any medication (the control group), the second group was treated with VA (standard VA group), and the third group was treated with *O*_1_ (optimized formulation group). All groups were fasted for 12 h, but were permitted free water intake. A dose of 10 mg/kg of VA suspended in water (standard VA) was administered to the VA group or a known volume of *O*_1_ equivalent to 10 mg/kg was administered to the vanillic formulation group [26]. The doses were administered orally, through an intragastric tube. Blood samples were collected via the retro-orbital plexus at times 10, 20, 30, 60, 90, 120, 360, 1440, and 2880 min, in heparinized tubes and immediately centrifuged at 13,000 × g for 10 min at 4 °C. The plasma was stored at − 80 °C till further analysis, by HPLC [27]. Pharmacokinetics parameters were calculated and analyzed by non-compartmental analysis using the Kinetica™ 2000 software (version 4.4.1 Thermo Electron Corporation, USA).

#### Pharmacodynamic study

- Induction of myocardial infarction
  Myocardial infarction (MI) was induced in rats by subcutaneous injection of 100 mg/kg isoprenaline hydrochloride dissolved in saline once daily, for two successive days. The dose and route of injection of isoprenaline hydrochloride were chosen based on previous literature [28, 29].
- Experimental design
  Rats were randomly allocated into 4 groups (*n* = 6). The first two groups received distilled water (p.o.) for 14 days and served as normal and myocardial infarction (MI) control groups, respectively. The third group received VA (10 mg/kg; p.o.), (VA group) for 14 days, while the last group received the same dose of VA in the optimized formulation (vanillic formulation group). All groups except the normal group received isoprenaline (100 mg/kg; s.c.) in the last 2 days of treatment. The dose of VA was chosen based on previous studies [26].
  After 24 h from the last injection of test agents, the animals were anesthetized with urethane (1.5 g/kg; i.p.) and subcutaneous peripheral limb electrodes were inserted for electrocardiographic (ECG) recording (Biocare ECG 101, USA). Rats were sacrificed by cervical dislocation, and blood samples were collected from the retro-orbital plexus. The hearts were rapidly isolated and used for the biochemical investigation of miR-217, mitogen-activated protein kinase (MAPK), phosphoinositide 3-kinase (PI3K), protein kinase-B (AKT), interleukin-6 (IL-6), tumor necrosis factor-α (TNF-α), reduced glutathione (GSH), and malondialdehyde (MDA), as well as the histological examination and immunohistochemical investigation of nuclear factor-κB (NF-κB).
- Biochemical assays
  - Determination of serum CK-MB activity and cardiac MDA and GSH contents
    The activity of creatine kinase-MB (CK-MB) in serum was determined using Stanbio CK-MB diagnostic kit (Boerne, TX, USA). The cardiac level of lipid peroxidation was measured as malondialdehyde (MDA), and the content of GSH was determined according to the manufacturer’s instructions using Biodiagnostics standard kit (Cairo, Egypt).
  - Quantitative RT-PCR analysis of miR-217, MAPK, and PI3K
    The rat hearts were used for isolation of the total RNA using Trizol (Invitrogen; Auckland, New Zealand), according to the manufacturer’s instructions. Reverse transcriptase M-MLV (Promega, Madison, WI, USA) was used to reverse-transcribed the isolated RNA into cDNA. The following primer sequences were used in the current experiment: for MAPK, forward primer sequence: 5′-CGAAATGACCGGCTACGTGG-3′, reverse primer sequence: 5′-CACTTCATCGTAGGTCAGGC-3′; for PI3K, forward primer sequence: 5′-CTCTCCTGTGCTGGCTACTGT-3′, reverse primer sequence: 5′-GCTCTCGGTTGATTCCAAACT-3′; for β-actin, forward primer sequence: 5′-CTGAGAGGGAAATCGTGCGT-3′, reverse primer sequence: 5′-TTGTTGGCATAGAGGTCTTTA -3′.
    Small RNA species-enriched RNA was isolated for miRNA quantitative reverse transcriptase PCR according to the manufacturer’s instructions (mirVana miRNA isolation kit; Ambion, Austin, TX, USA). miRNA was reverse-transcribed by using Ncode miRNA first-strand complementary DNA synthesis kits (Invitrogen; Auckland, New Zealand). Forward primer sequence was designed as the corresponding mature miRNA sequences and U6 snRNA (forward primer sequence: 5′-CTCGCTTCGGCAGCACATATACT-3′ and reverse primer sequence: 5′- ACGCTTCACGAATTTGCGTGTC-3′) were used as normalizing control. The miR-217 specific primers; forward primer sequence: 5′-TACTGCATCAGGAACTGACTGGA-3′ and reverse primer 5′-GTGCAGGGTCCGAGGT-3′.
    Quantitative reverse transcriptase PCR was performed by using a Power SYBR Green PCR Master Mix on the CFX96 Instrument (Bio-Rad, USA). Data analysis was determined by using the relative standard curve method.
  - ELISA assay of IL-6 and AKT
    The cardiac contents of IL-6 and AKT were measured by enzyme-linked immunoassay (ELISA) technique using a standard kit (Cloud-clone corp; TX., USA).
  - Western blot analysis of TNF-α
    Part of the heart was homogenized using radioimmunoprecipitation assay (RIPA) buffer (50 mM Tris HCl pH 8, 150 mM NaCl, 1% Triton X-100, 0.5% sodium deoxycholate, and 0.1% SDS) provided with phosphatase inhibitor cocktail. After protein quantification according to the manufacturer’s instruction (ThermoFisher Scientific, MA, USA), 7.5 μg of protein from each sample was loaded on gel electrophoresis and transferred to the PVDF membrane. The membrane was blocked with 5% bovine serum albumin and incubated overnight (1:1000; 4 °C) with either anti-TNF-α or anti-β-actin antibody (ThermoFisher Scientific, MA, USA). Chemiluminescence detection was performed with an Amersham detection kit according to the manufacturer’s protocols and exposed to X-ray film. Protein was calculated using densitometric analysis of the autoradiograms by a scanning laser densitometer (GS-800 system, Bio-Rad, CA, USA). Results were expressed as arbitrary units after normalization for β-actin protein expression [30].
  - Histopathological assessment of myocardial damage
    Autopsy samples of hearts were taken from the different groups and fixed in 10% formalin. Washing was done using tap water; then, for dehydration, serial dilutions of alcohol were used. Specimens were cleared in xylene and embedded in paraffin for 24 h. Paraffin beeswax tissue blocks were prepared for sectioning at 4 microns using a slide microtome. The obtained tissue sections were collected on glass slides, deparaffinized, and stained by hematoxylin and eosin (H&E) stains for histopathological examination using the electric light microscope [31].
  - Immunohistochemical reaction of NF-κB in heart tissue
    Heart tissue sections embedded in paraffin (around 3 µm thickness) were used for detection of NF-κB through the immunostaining with primary antibody polyclonal immunoglobulin-G of rat NF-κB according the method previously described by Zaafan et al. [32]. Finally, grading of immunohistochemical reactivity was measured from 4 randomly chosen fields in each section and averaged using image analysis software (ImageJ, Fiji version; MD, USA).

## Statistical analysis

Data were analyzed using the Design-Expert 13.0.5.0 software (Stat-Ease Inc., USA), and was used for the optimization step after analysis of variance (ANOVA).

Data from pharmacokinetic and pharmacodynamic studies were presented in the form of mean ± SEM. The comparisons among means of different groups were done via one-way analysis of variance (ANOVA) and Tukey–Kramer multiple comparisons posttest. Kruskel–Wallis test was used for analyzing the histopathological scores and followed by Dunn’s multiple comparisons test. The level of significance was taken as *p* < 0.05. The statistical tests were carried out using the GraphPad Prism software package, version 5 (GraphPad Software, Inc., USA).

## Results and discussion

The VA-PC complex was successfully prepared by reflux in tetrahydrofuran, which was confirmed by XRD, IR, and DSC. The formed complexes were further hydrated to allow the formation of the self-assembled vesicles [15].

### Drug content analysis

Pharmacosomes showed a high drug content of greater than 97% for all formulations (results not shown). This indicates the uniform complexation and binding of VA with phosphatidylcholine, allowing the drug delivery to be clinically feasible [17]. Many vesicular systems, such as liposomes and niosomes, require special techniques as surface modification, to improve the loading of the drug, but with pharmacosomes, there is no need for such treatment, as the drug is reversibly bonded with the phospholipids. This would result in an improvement in the drug loading and also enhancement of the system’s stability [33].

### Vanillic acid-phosphatidylcholine interaction

#### Infrared spectroscopy

**Fig. 1:**
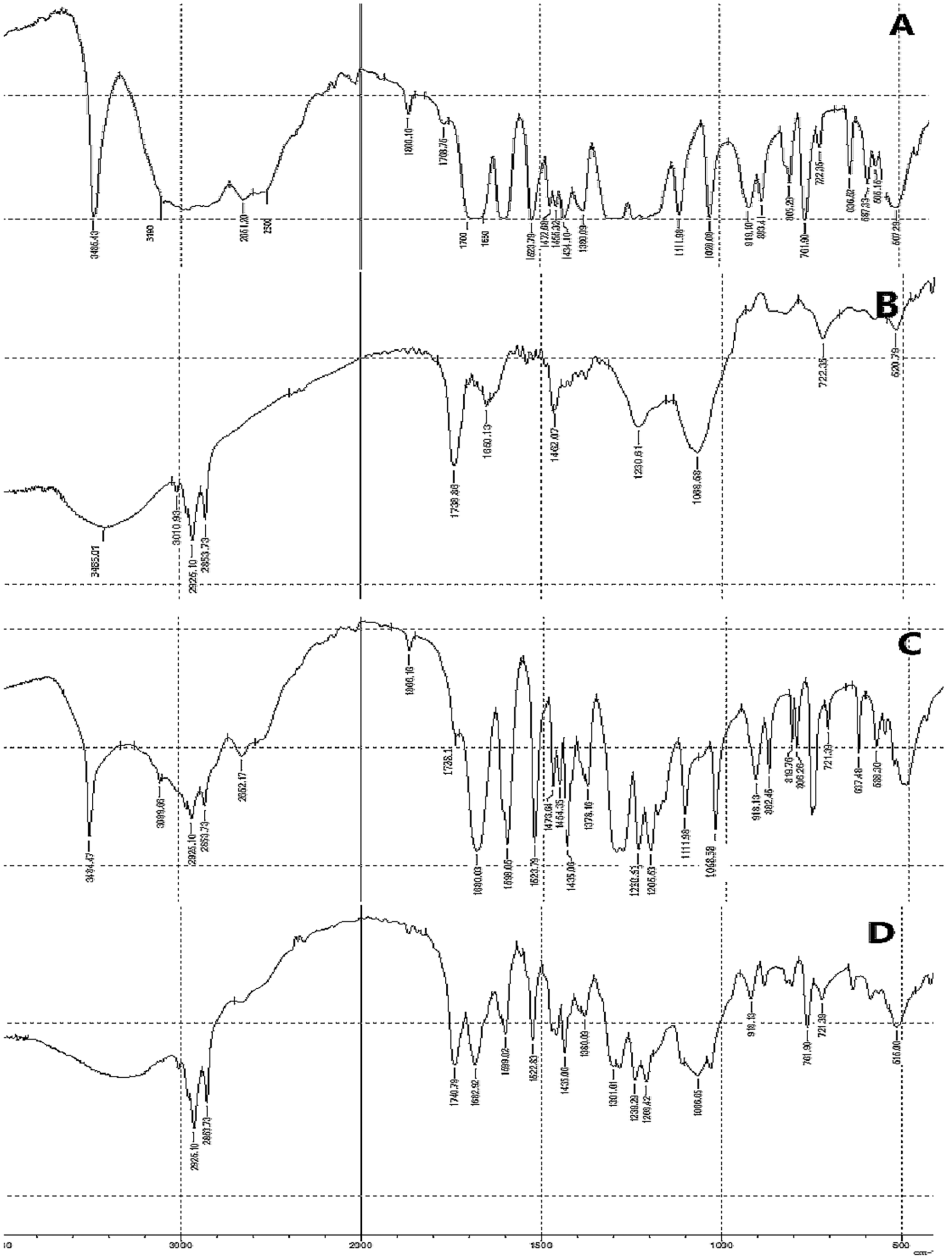
IR spectrum of **A** vanillic acid, **B** phosphatidylcholine, **C** physical mixture, and **D** VA-PC complex

Infrared spectroscopy was used to confirm the formation of the complex. The spectrum of the formed complex was compared with the individual components and their corresponding physical mixture, as shown in Fig. 1. A significant difference was observed between the complex and the physical mixture. As can be observed that VA (Fig. 1A) showed a characteristic broad band stretching at 3485.43 cm^−1^; corresponding to the phenolic hydroxyl groups, and a broad band from 2500 to 3190 cm^−1^ corresponding to the carboxylic hydroxyl group. Moreover, an intense band from 1700 to 1650 cm^−1^, appeared with VA, which corresponds to the carbonyl stretch C = O of carboxylic acid.

Characteristic peaks were present in phosphatidylcholine (Fig. 1B), which were 3485.01 cm^−1^ of the phenolic hydroxyl group, 2925.1 cm^−1^ of the -CH_2_ group, and several peaks below 1800 cm^−1^, such as 1738.1 cm^−1^ of the C = O group, 1230.61 cm^−1^ of the P = O group, and 1068.58 cm^−1^ of the P-O-C group.

The physical mixture showed the same peaks that were present in VA and PC as shown in Fig. 1C. On the contrary, the phenolic hydroxyl group was shifted to a lower frequency, and broadening of the peak was observed as represented in Fig. 1D. The complex showed bending at 3200 − 3600 cm^−1^, which indicates that VA might have reacted with PC. Furthermore, C = O, P = O, and P-O-C groups were shifted in the complex to 1740.79, 1239.9, and 1066.65 cm^−1^ respectively. All these indicate the existence of an interaction between VA and PC. This interaction is probably a weak hydrogen bond or van der Waals force, which was formed between the acidic hydroxyl group of the VA and the phosphate group of the PC [20].

#### X-ray diffractometry

**Fig. 2:**
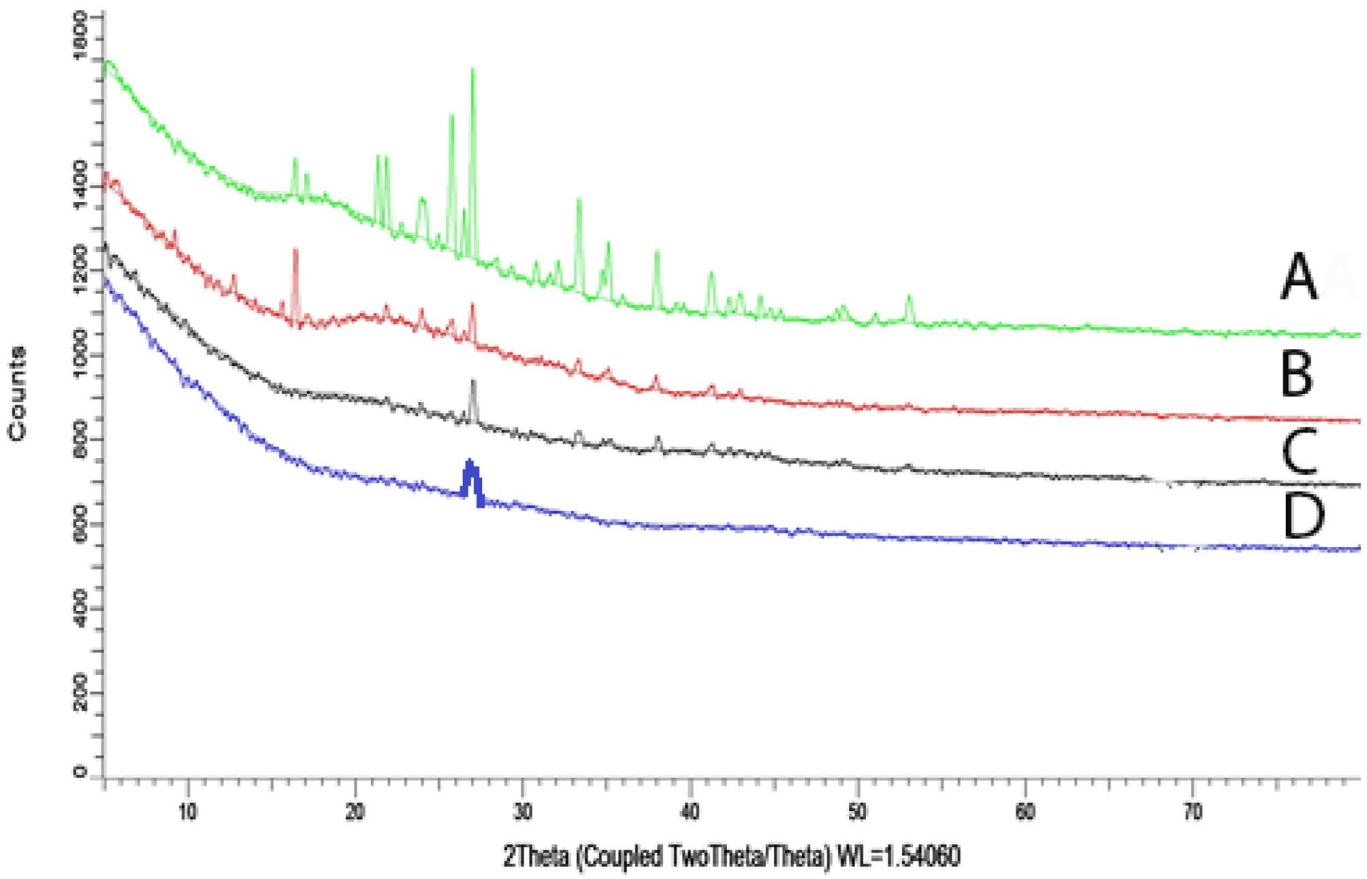
Diffractogram of **A** vanillic acid, **B** physical mixture, **C** VA-PC complex and **D** phoshatidyl choline

XRD is used to determine the physical state of VA and to confirm the complexation that may occur with the phospholipid [22]. As indicated by the diffractograms represented in Fig. 2, the diffraction pattern of VA showed characteristic distinctive peaks, confirming its crystalline structure. On the other hand, the amorphous structure of PC was confirmed by the absence of peaks in its diffraction pattern. The diffraction pattern of the physical mixture showed some of the characteristic peaks of the drug, whereas the complex showed an almost complete disappearance of the characteristic peaks of the drug, with the appearance of the characteristic peak of the PC. This confirms the formation of the complex, where VA lost its crystalline structure, and either transformed to its amorphous form or was molecularly dispersed [34].

#### Differential scanning calorimetry

The thermal behavior of many compounds can be analyzed using differential scanning calorimetry. The changes in the material are measured as a function of controlled changes in temperature. DSC provides useful information about melting, degradation, compatibility, stability, and interaction. These changes are expressed as enthalpy changes, with the change in peaks and peaks’ onset time and shape, with the relative area [34].

**Fig. 3:**
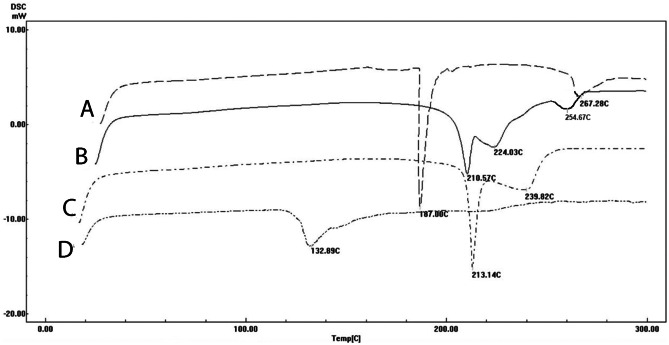
DSC thermogram of **A** phosphatidylcholine, **B** physical mixture, **C** vanillic acid, and **D** VA-PC complex

As can be observed from Fig. 3A, PC showed a sharp endothermic peak at 187 °C, probably due to the transition from gel to liquid crystal state, where the non-polar hydrocarbon tail of PC might be melted. This melting probably occurred in 2 phases, giving rise to a smaller broad peak at 267.28 °C, [35]. VA showed a characteristic sharp endothermic peak at 213.4 °C (Fig. 3C), with a small broad peak at 239.81 °C, confirming its crystalline nature. The physical mixture (Fig. 3B) showed two endothermal peaks at 210.57 °C, 224.03 °C that correspond to VA, and a third peak at 254.67 °C that corresponds to PC. It is worth noting that the other peak of the PC was not detectable in the physical mixture. Similar results were obtained by Das and Kalita [14]. This could be attributed to the controlled increase in the temperature, which can cause melting of the individual components, with partial formation of aggregation in situ, resulting in a slight change in the melting points than the individual ingredients [34]. On the other hand, the thermogram of the VA-PC complex (Fig. 3D), showed the disappearance of those characteristic peaks, and the appearance of a new phase transition temperature at 132.89 °C, which is completely different from the peaks of the individual components. It is assumed that the heat used in the formation of the complex resulted in the melting of the lipid, and dissolution of the drug, with a formation of the complex, through hydrogen bond or van der Waal’s forces. The interaction between VA and the polar parts of PC, made the carbon-hydrogen chain of the PC turn freely and enwrap the PC polar parts, resulting in a decrease in the sequence between the PC aliphatic hydrocarbon chains, causing the endothermal peak of the PC to disappear, and to lower the phase transition temperature and to appear at 132.89 °C [20].

### Central composite design analysis

The central composite design was the design of choice for the response surface methodology in the experimental design as it has the capability of creating orthogonal blocks, allowing model terms and block effects to be estimated independently and minimizing the variation in the regression coefficients [36]. Moreover, it allows to approximate the response function of experimental data with the quadratic functions [37, 38].

As shown in Table 2, the ANOVA results of the studied factors showed a high correlation coefficient *R*^2^ for all the studied factors, with a reasonable agreement between the predicted *R*^2^ and the adjusted *R*^2^, and an adequate precision, of a value greater than 4. This indicates that the model could be used to navigate the design space [39].

**Table 2:** ANOVA results for the studied factors

**Source**	**PS**					**PDI**						**ZP**				
	**Sum of squares**	**df**	**Mean square**	***F* value**	***p*-value prob > *F***	**Sum of squares**		**df**	**Mean square**	***F* value**	***p*-value prob > *F***	**Sum of squares**	**df**	**Mean square**	**F value**	***p*-value prob > *F***
**Order**	Quadratic					Quadratic						Quadratic				
**Model**	47303.8	5	9460.8	68.2	< 0.0001	0.11		5	0.021	17.1	0.0008	518.34	5	103.67	65.29	< 0.0001
***X***_**1**_**—*PC:VA***	*3672.8*	*1*	*3672.8*	*26.5*	*0.0013*	*0.019*		*1*	*0.019*	*15.6*	*0.0055*	*367.75*	*1*	*367.75*	*231.61*	< *0.0001*
***X***_**2**_**—*Prec.conc***	*89.4*	*1*	*89.39*	*0.6*	*0.4485*	*4.2E − 003*		*1*	*4.225E − 003*	*3.4*	*0.1068*	*48.40*	*1*	*48.40*	*30.48*	*0.0009*
***X***_**12**_	*0.5*	*1*	*0.49*	*3.5E − 003*	*0.9543*	*1.3E − 017*		*1*	*1.3E − 017*	*1.2E − 014*	*1.0000*	*0.016*	*1*	*0.016*	*9.8E − 003*	*0.9238*
***X***_**1**_^**2**^	*25257.9*	*1*	*25257.9*	*182.1*	< *0.0001*	*0.025*		*1*	*0.025*	*19.9*	*0.0029*	*3.20*	*1*	*3.20*	*2.01*	*0.1987*
***X***_**2**_^**2**^	*12843.9*	*1*	*12843.9*	*92.6*	< *0.0001*	*0.047*		*1*	*0.047*	*38.3*	*0.0004*	*101.94*	*1*	*101.94*	*64.20*	< *0.0001*
**Residual**	970.8	7	138.7			8.6E* − *003		7	1.2E* − *003			11.11	7	1.59		
***Lack of fit***	*760.8*	*3*	*253.6*	*4.8*	*0.0811*	*6.1E − 003*		*3*	*2.0E − 003*	*3.2*	*0.1483*	*8.23*	*3*	*2.74*	*3.81*	*0.1144*
***Pure error***	*209.9*	*4*	*52.5*			*2.6E − 003*		*4*	*6.4E − 004*			*2.88*	*4*	*0.72*		
**Cor total**	48274.6	12				8.6E* − *003		7	1.2E* − *003			529.45	12			
***R***^**2**^		0.9799				0.9244						0.9790				
**Adjusted *R***^**2**^		0.9655				0.8705						0.9640				
**Predicted *R***^**2**^		0.8811				0.6873						0.8809				
**Adequate precision**		30.183				16.164						25.747				

#### Particle size analysis

Vesicular size plays a vital role in a successful drug delivery system, as particles greater than 400 nm in size are known to be identified by the reticuloendothelial system (RES), leading to their short half-life in the blood [40]. Particle size ranged from 168.5 ± 4.69 to 416.3 ± 2.67 nm, as shown in Table 1. This particle size range might have a great role in sustaining the release and enhancing the oral drug bioavailability [34].

The relationship between the particle size and the studied factors is presented in Eq. 4.

4 $$\mathrm{PS}=+316.60-21.43*{X}_{1}+3.34*{X}_{12}-60.26*{X}_{1}^{2}+42.97*{X}_{2}^{2}$$

**Fig. 4:**
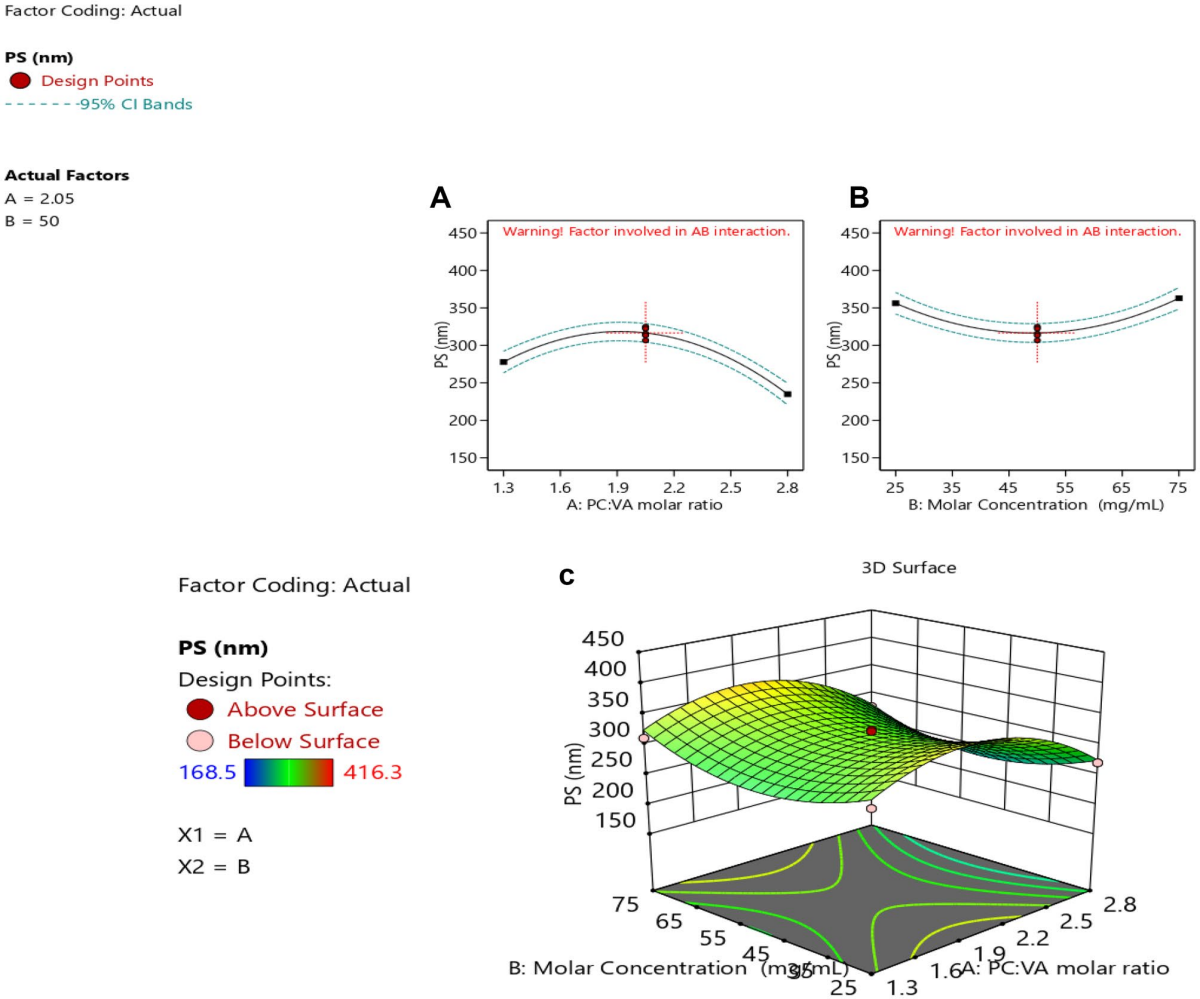
Response surface plots of particle size **A** quadratic effect of the PC:VA molar ratio on PS, **B** quadratic effect of the precursor concentration on PS, and **C** 3-D response surface plot of the combined studied factors on the PS

Further analysis using ANOVA as presented in Table 2 shows that the studied factors had a significant effect on the particle size, except the individual effect of precursor concentration (*X*_2_). The model was found to be quadratic as observed from Fig. 4A and Table 2. As can be observed, increasing the PC:VA resulted in an initial increase in the PS. This could be attributed to the formation of micellar structure, as the PC amount increases, which could have a small size, when PC was present in small amounts. On the other hand, increasing the PC amount resulted in the formation of vesicles, which could have a larger size [17]. Moreover, increasing the PC:VA leads to less availability of VA, for the complex formation, which would result in an increase in the vesicular size. These results were in accordance with Das and Kalita [14]. This effect was observed when using a ratio of PC:VA up to 2:1.

A further increase in the PC:VA ratio (greater than 2:1) resulted in a decline in the vesicular size. This could be attributed to the increase in the viscosity of the medium at the high PC amount, which could prevent the agglomeration of the pharmacosomes, and hence, smaller sizes of vesicles might be produced [41, 42].

As can be depicted from Table 2, a quadratic effect existed between the PS and the precursor concentration as shown in Fig. 4B. An initial reduction in the particle size was observed by increasing the precursor concentration, which was followed by an increase in the PS, by the further increase in the precursor concentration. This could be related to the effect of the precursor concentration on the nucleation rate of nanoparticle formation and supersaturation. The initial decrease in the vesicular size with increasing the precursor concentration (till 50 mg/mL) could be due to the increase in the nuclei formation, by increasing the total precursor concentration, where a larger number of nuclei make smaller particles [43].

A further increase in the precursor concentration (> 50 mg/mL) resulted in an increase in the vesicular size. This could be attributed to the higher rate of crystal growth at higher concentrations, resulting in bigger vesicular sizes [44].

A 3-D response surface plot, showing the effect of both factors on the particle size, shows a non-linear relationship as represented in Fig. 4C. An initial increase in the particle size was observed by increasing the PC:VA molar ratio, together with reducing the precursor concentration, which was followed by a decrease in the vesicular size with a further increase in PC:VA together with lowering the precursor concentration.

#### Polydispersity index analysis

It is well known that the particle size homogeneity has a major role in the fate of the nanoparticles [22], and therefore, it was deeply explored in the CCD.

The PDI of all the prepared formulations ranged from 0.25 ± 0.03 to 0.66 ± 0.01, as shown in Table 1.

The relationship between the PDI and the studied factors is shown in Eq. 5

5 $$\begin{aligned}\mathrm{PDI}=&\;+0.42+0.049*{X}_{1}+0.023*{X}_{2}+0.000*{X}_{12}\\&-0.060*{X}_{1}^{2}+0.082*{X}_{2}^{2}\end{aligned}$$

**Fig. 5:**
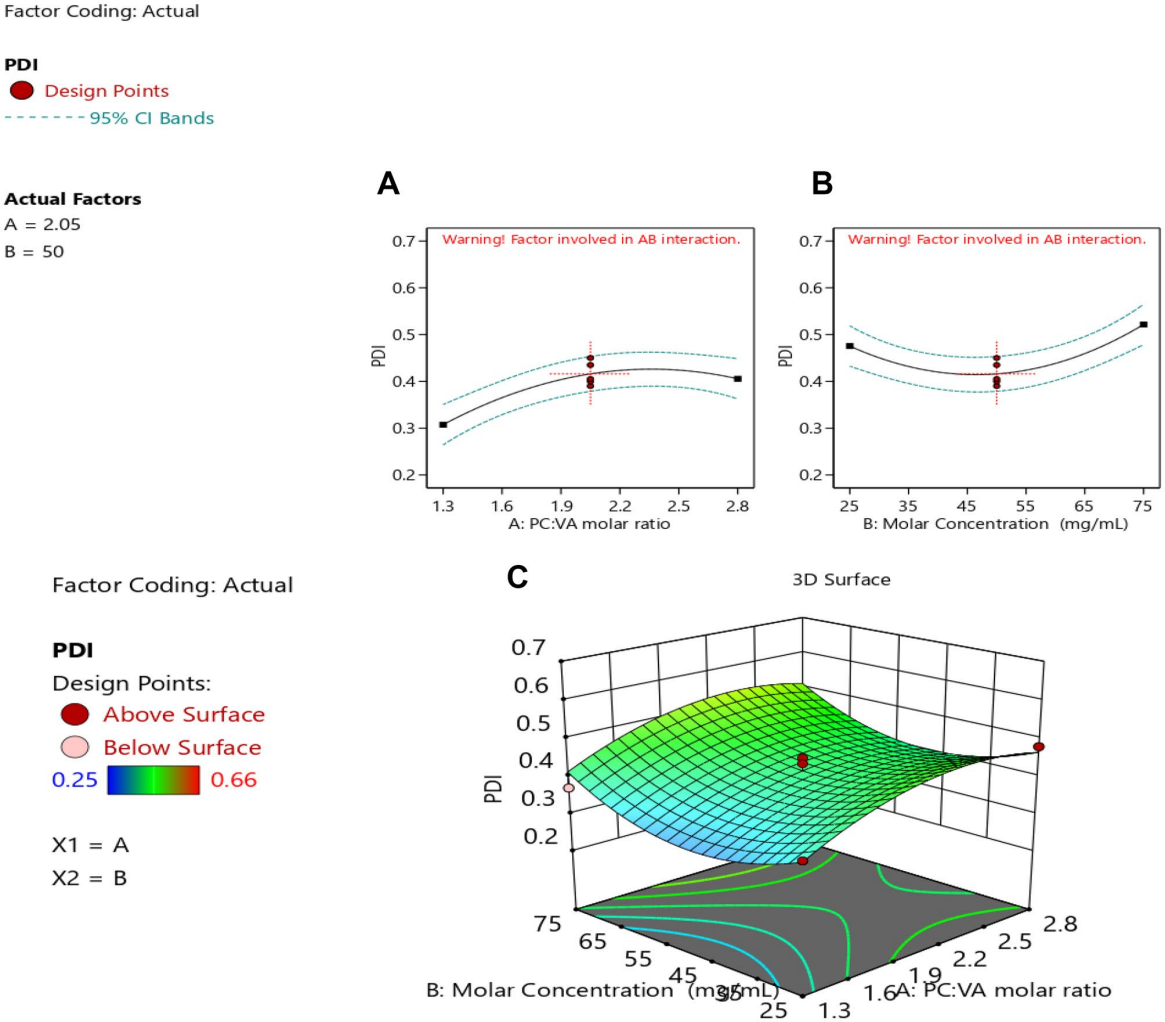
Response surface plots of PDI **A** quadratic effect of the PC:VA molar ratio on PDI, **B** quadratic effect of the precursor concentration on PDI, and **C** 3-D response surface plot of the combined studied factors on PDI

ANOVA was used for further analysis of the PDI, as shown in Table 2. As can be observed, the model was found to be quadratic. Figure 5 A shows the effect of the PC:VA molar ratio on the PDI, where it showed a biphasic effect on the PDI. Increasing the PC:VA up to a ratio of 2:1 resulted in an initial increase in the PDI, whereas a further increase resulted in a smaller vesicular size. The initial increase in the PDI could be attributed to the less complex formation caused by the increase in PC relative to VA, as a result of increasing the PC:VA [17]. Increasing the PC:VA more than 2:1 may result in an increase in the viscosity of the medium. Consequently, the agglomeration of the particles would be reduced, thus reducing the heterogeneity and the PDI [41].

It has been observed that increasing the precursor concentration resulted in a smaller PDI, till 50 mg/mL; this could be due to the increase in the nuclei formation by increasing the precursor concentration, with the consequence of the formation of homogenous small particles, whereas at a concentration above 50 mg/mL, a higher rate of crystal growth to some particles might have occurred, resulting in an increase in the system heterogenicity [43].

Increasing the precursor concentration to 50 mg/mL resulted in an initial decrease in the PDI, which was followed by an increase in the PDI, as shown in Fig. 5B.

The 3-D response surface plot as illustrated in Fig. 5C, shows that the PDI was found to decrease initially by decreasing the PC:VA together with increasing the precursor concentration. By further decreasing PC:VA and increasing the precursor concentration, the PDI was increased.

#### Zeta potential analysis

Zeta potential is considered a crucial factor for indicating the stability of any colloidal system. An absolute zeta potential of 20 to 30 mV, indicates flocculation, that overcomes the repulsion forces between the particles [34]. Moreover, the ZP can predict the fate of the nanoparticles in vivo [22]. In the present study, the zeta potential ranged from − 19.0 ± 0.89 to − 39.2 ± 0.63 mV. The high negative value of the zeta potential could be attributed to the presence of PC in a neutral medium, resulting in a negative charge on the pharmacosmes’ surface [22].

The relationship between the ZP and the studied factors is shown in Eq. 6.

6 $$\begin{aligned}\mathrm{ZP}=&\;+32.20+6.78*{X}_{1}+2.46*{X}_{2}-0.063*{X}_{12}\\&-0.68*{X}_{1}^{2}-3.83*{X}_{2}^{2}\end{aligned}$$

**Fig. 6:**
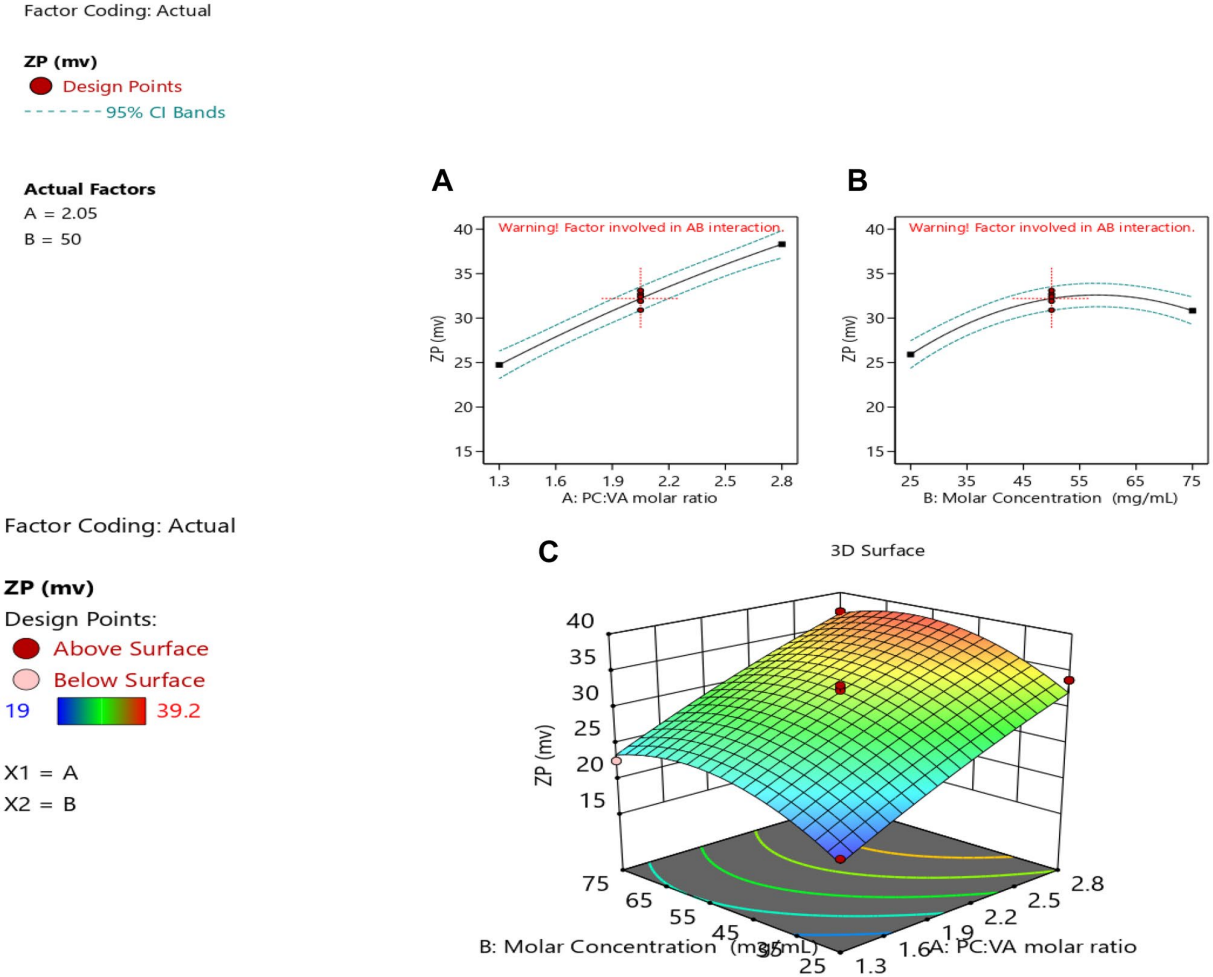
Response surface plots of ZP **A** quadratic effect of the PC:VA molar ratio on ZP, **B** quadratic effect of the precursor concentration on ZP, and **C** 3-D response surface plot of the combined studied factors on ZP

Further analysis using ANOVA (Table 2) shows a quadratic effect between the ZP and the studied factors. As seen in Fig. 6A, ZP increased upon increasing the PC:VA ratio. This could be due to the increase of phosphatidylcholine and the decrease of VA, which resulted in less complexation between the drug and the phosphate group of the PC. Thus, the negatively charged phosphate group will be more available, imparting more charges on the surface of vesicles [45].

Increasing the precursor concentration up to 50 mg/mL had a positive impact on the ZP, as shown in Fig. 6B. This could be due to the increase in the formation of vesicles, by increasing the precursor concentration greater than 50 mg/mL, carrying negative charges on their surface. However, a further increase in the precursor concentration did not affect the ZP [46]. This is probably due to the saturation of the medium with the vesicles at that point. Moreover, increasing the precursor concentration resulted in increasing the viscosity which hindered the formation of more vesicles [47].

The 3-D response surface plot represented in Fig. 6C which shows an increase in the ZP upon increasing both the PC:VA molar and the precursor concentration.

#### Optimization using central composite design

A new optimized formulation was selected based on the desirability approach, using a numerical optimization technique. The optimized formulation (*O*_1_) was selected to have a minimal particle size, and PDI together with the maximum ZP [48]. Accordingly, the optimized formulation; as suggested by the software was prepared and evaluated in terms of the aforementioned tests, as represented in Table 3, with a desirability of 0.522. The observed results of the prepared formulation were compared to the expected results, to calculate the % bias, in order to validate the design, and to indicate the robustness of the model [34]. As can be observed the values of % bias for all studied responses was less than 25%, indicating the validity of the design [49].

**Table 3:** Optimized formulation as suggested by software together with the expected and observed results

	**PC:VA molar ratio**	**Precursor concentration (mg/mL)**	**PS (nm)**	**PDI**	**ZP (mV)**
**Expected result**	1.3: 1	53.164	278.928	0.312	-25
**Observed result**			229.7 ± 3.76	0.29 ± 0.07	-30.8 ± 1.87
*%bias			21.43%	7.05%	23.20%

A small-sized particle (229.7 ± 3.76 nm), with a narrow size distribution of (0.29 ± 0.07), together with a high zeta potential value (-30.8 ± 1.87) suggests a suitable nanoparticle system, which escapes the RES, and helps in reaching the site of action. The optimized formula (*O*_1_) was further characterized to ensure its robustness and efficacy.

$$*\% \, \mathrm{bias}=\frac{(|\mathrm{Expected }-\mathrm{ Observed}|)}{\mathrm{Expected}}*100$$

### Morphology of pharmacosomes vesicles

The surface morphology of the optimized formulation was examined using a scanning electron microscope, whereas a transmission electron microscope was used to examine the aggregates.

#### Scanning electron microscope

**Fig. 7:**
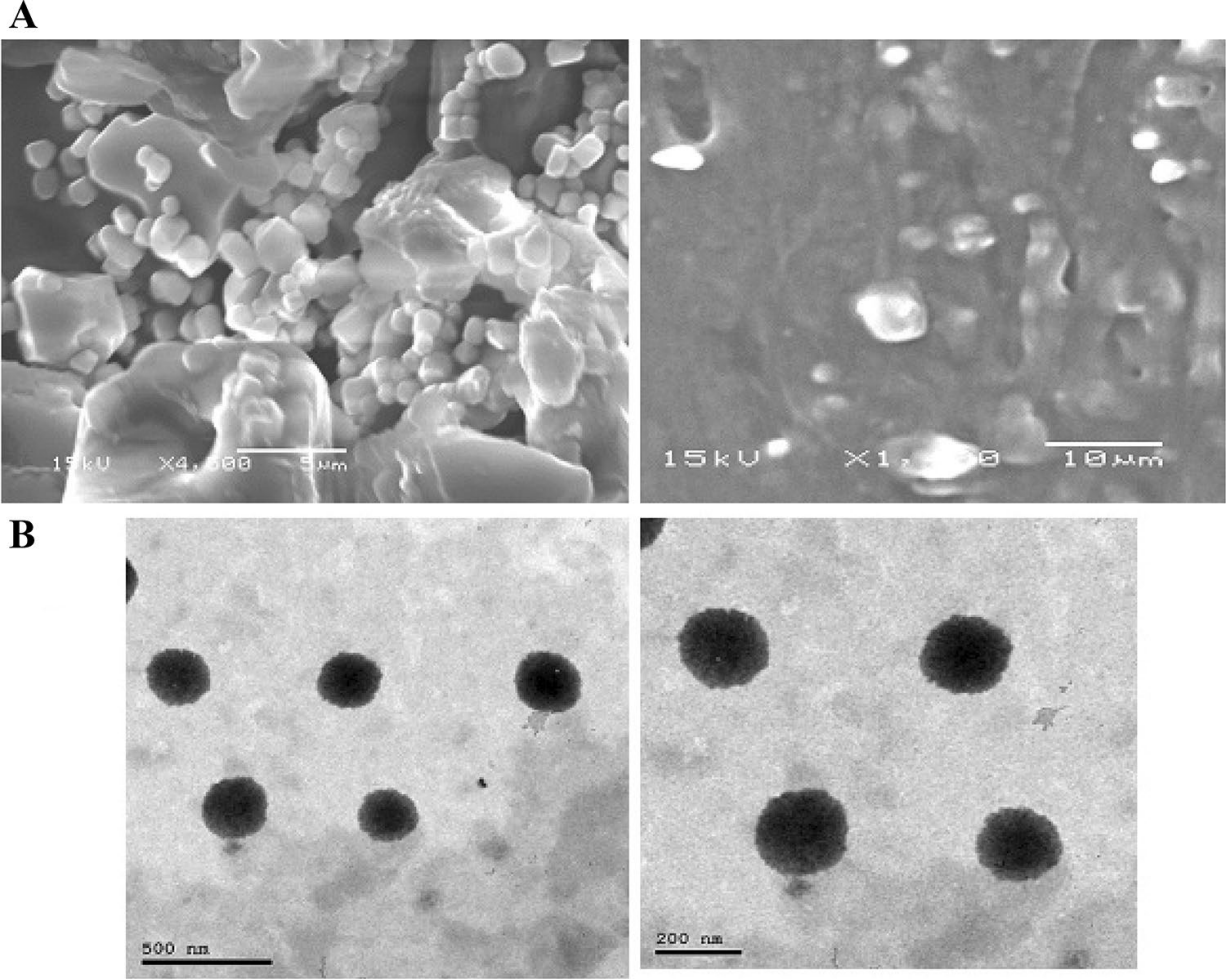
Morphological structure of pharmacosomes. **A** Scanning electron microscope image of VA-loaded pharmacosomes. **B** TEM image of VA-loaded pharmacosomes

Scanning electron microscope micrographs as represented in Fig. 7A shows the surface morphology of the pharmacosomes. The pharmacosomes appeared to be slightly spherical in shape.

#### Transmission electron microscope

The aggregates of the pharmacosomal dispersion were characterized using TEM, as represented in Fig. 7B. TEM micrographs of pharmacosomes show unilamellar vesicles, having a liposome-like structure, with uniform size. The polar groups of the PC were complexed with VA, probably by hydrogen bonding, to get a well-defined vesicular structure for VA delivery. When the complex was hydrated with an aqueous medium, the vesicles were arranged in response to surface tension [22].

### Solubility and partition coefficient

Table 4 shows the saturated solubility of VA and its improvement by complexation into pharmacosomes. The increase in the water solubility of the optimized formulation as compared to that of VA could be attributed to the micellar formation, resulting in the solubilization of VA [50]. Moreover, the amphiphilic nature of the complex may enhance wetting and dispersibility, which in turn may also enhance bioavailability [33].

**Table 4:** Solubility and LogP of vanillic acid and the optimized formulation

**Solubility**	**VA**	**VA-loaded pharmacosomes**
**Water**	543.9 µg/ml	1053.5 µg/ml
**n-Octanol**	1948.8 µg/ml	29880 µg/ml
**Partition coefficient**	3.58	28.36
**Log P**	0.553	1.45

The optimized formulation resulted in an improvement in the partition coefficient (8 times), together with Log P (3 times), as seen in Table 4. This proves that the optimized formulation enhanced the aqueous solubility, as well as the lipophilicity of VA. This may be due to the masking of the polar group of the VA in the pharmacosomes [20].

The optimized formulation showed a Log P value of 1.45, which is considered excellent for oral drug delivery according to Lipinski’s Rule of Five, where he identified the ideal value for Log P to be from 1.35–1.8 [51].

The optimized formulation showed a Log P value of 1.45, which is an ideal value according to Lipinski's Rule of Five, where he identified the ideal value for Log P to be from 1.35–1.8. This value is considered excellent for oral drug delivery absorption [51].

### In vitro drug release

**Fig. 8:**
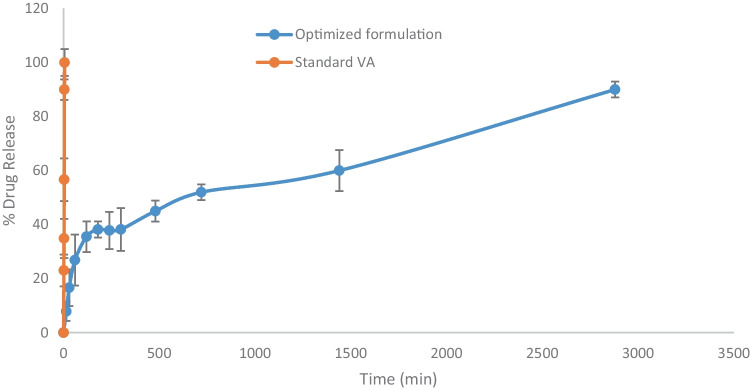
In vitro drug release profile of VA from standard and optimized formulation

The in vitro release profile of VA from standard VA was compared with the optimized formulation. As can be observed from Fig. 8, the VA-PC complex in the optimized formulation showed an enhanced release of VA as compared to standard VA, at 4 h, which corresponds to about 40% of the drug released. This is probably due to the enhancement of the dissolution of VA when incorporated with PC in the complex. PC is an amphiphilic surfactant that can enhance the solubility of the drug by the action of wetting and dispersion The in vitro release also indicated a sustained release after 4 h for the optimized formulation as compared with the standard VA. The standard drug was almost completely released after 6 h,whereas the release of the VA from the optimized formulation followed a biphasic pattern, which is characterized by an initial burst release within the first 4 h probably due to the presence of the VA absorbed at the surface of the vesicles. The initial burst release was followed by a sustained release up to 48 h, which could be attributed to the presence of the VA-PC stable complex within the vesicles, which is homogenously associated within the space of the vesicles. The slow release of VA from the pharmacosome vesicles could be attributed to 2 stages, the dissociation of VA from the complex, and the release of standard VA throughout the vesicular structure [22].

The in vitro release profile of VA from the optimized formulation indicates the suitability of the pharmacosomes for enhancing the solubility of the drug and controlling its release.

### Stability study of pharmacosomes

The safety and efficacy of drug products are assured by applying in vitro stability studies [22]. The results of the short-term stability; over a 3-month period at 4 °C showed no significant change in the drug content, a PS of 227.89 ± 6.98 nm with PDI of 0.27 ± 0.03 and a ZP of 29.92 ± 1.90 mV, which were not statistically significant than when prepared (*p* value < 0.0001). Moreover, no color change or appearance of aggregates was observed visually. The results indicate the excellent stability of the prepared pharmacosomal vesicles at 4 °C.

### In vivo pharmacokinetic study

#### HPLC method development and optimization

**Fig. 9:**
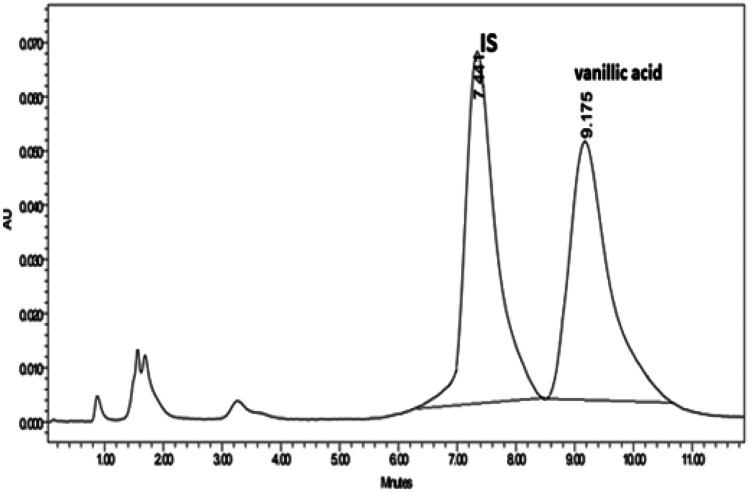
HPLC chromatogram showing separated peaks of VA and IS

The quantitation of minute amounts of the drug in plasma and their efficient extraction from the plasma matrix with acceptable recovery represent a challenge in bioanalysis. Two C_18_ columns with different lengths (100 mm and 250 mm) were tested for this elution, where the shorter one showed an acceptable resolution between VA and internal standard and shorter runtime. Several mobile phase compositions were tested including different ratios of solvents like acetonitrile, methanol, phosphate buffer, and acetate buffer. Finally, Kinetex C_18_ (4.6 mm ID × 100 mm L, particle size 5 µm, Phenomenex, USA) was used with mobile phase composition consisting of 20% methanol and 80% acetic acid (0.1%), at UV detection at 272 nm. The chromatogram showed sharp and symmetric resolved peaks with good sensitivity of the analyte (Fig. 9). The developed methods have the advantage of using a greener mobile phase than the other reported methods, where elution was carried out in a simple isocratic program with 20% organic solvent only [52, 53].

#### Validation sheet and extraction procedure

Simple protein precipitation was applied as it requires a small sample size and consumes a small quantity of organic solvents, thus reducing the cost and time. Methanol showed acceptable extraction recovery and matrix effect for VA from plasma samples as shown in Table 5. The stability studies showed acceptable results. A validation sheet is listed in Table 5, where the formic acid was added to improve the symmetry of the eluted peaks, and system suitability parameters were calculated according to the USP reference [54].

**Table 5:** System suitability and validation parameters for the proposed HPLC–UV method

**Parameters**	**HPLC**	**USP Reference values**
**Linearity (µg/mL)**	1–200	
**Correlation coefficient (*r*)**	0.9999	
**Slope**	2.291	
**Intercept**	0.7938	
**SD of residuals from line**	0.2201	
**LOD (µg/mL)**	0.317	
**LOQ (µg/mL)**	0.961	
**Accuracy (recovery % ± SD)**	101.34 ± 0.88	
**Precision (intraday)**	99.65 ± 1.65	
**Precision (inter-day)**	101.45 ± 1.98	
**Extraction recovery %**	7.554	
**Matrix effect**	8.423	
***t***_***R***_**, min**	9.18 ± 0.05	
**Tailing factor (*T*)**	1.04	*T* ≤ 2, *T* = 1 for symmetric peak
**Capacity factor (*K′*)**	2.11	*K'* = 1–10 acceptable
**Plates number (*N*)**	5884	*N* > 2000
**Height equivalent to theoretical plate (HETP)**	0.06	The smaller the value, the higher the column efficiency
**Experimental resolution (*R***_***s***_)**	2.08	*R*_s_ ≥ 2

**Experimental resolution between VA and internal standard

#### Pharmacokinetic study analysis

**Fig. 10:**
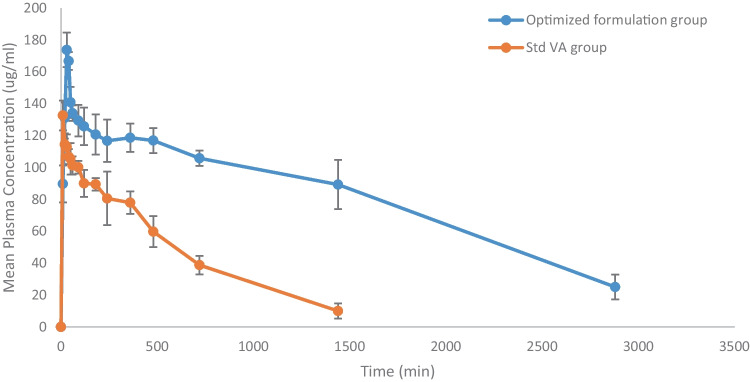
Mean plasma concentration–time curve of VA after oral administration. Data is presented as mean ± SEM

The oral bioavailability of VA from the optimized formulation was investigated in rats and compared to that of standard VA. The key pharmacokinetic parameters are summarized in Table 6, and the mean VA concentrations in rat plasma at different time intervals were plotted as illustrated in Fig. 10. After oral administration, VA from the optimized formulation was absorbed more slowly than standard VA, where the *T*_max_ values of the optimized formulation and standard VA groups were 30 h and 10 h, respectively. The peak plasma level of VA from the optimized formulation was much higher than that of standard VA, with *C*_max_ values being 173.72 μg/mL and 132.65 μg/mL, respectively.

**Table 6:** Pharmacokinetic parameters of standard VA and VA form the optimized formulation

	**Standard Vanillic acid**	**VA from Pharmacosomes**
***C***_**max**_** (μg/mL)**	132.65 ± 9.29	173.72 ± 10.78^a^
***T***_**max**_** (min)**	10 ± 2.56	30 ± 2.97^a^
**AUC**_**0–24**_** (μg/min/mL)**	67229.20 ± 3173.0	227687 ± 2365^a^
**AUC**_**0–***∞*_ **(μg/min/mL)**	72517.20 ± 7980.0	278594.41 ± 23760.0^a^
**AUMC**_**0–*t***_** (μg/min**^**2**^**/mL)**	31061100 ± 56800.0	250889000 ± 43870.0^a^
**AUMC**_**0–*∞***_** (μg/min**^**2**^**/mL)**	41480100 ± 34870.0	486900000 ± 34900.0^a^
**MRT (min)**	572 ± 6.98	1747.71 ± 28.7^a^

*C*_*max*_ peak plasma concentration, *T*_*max*_ time to reach peak plasma concentration, *AUC*_*0–24*_ area under the plasma concentration–time curve from time 0 to 24 h, *AUC*_*0–∞*_ area under the plasma concentration–time curve calculated by the linear trapezoidal rule from time 0 to infinity, *AUMC*_*0–24*_ area under the first moment curve from time 0 to 24 h, *AUMC*_*0–∞*_ area under the first moment curve from time 0 to infinity

^a^Significant difference from standard vanillic acid group. Data is presented as mean ± SEM

The mean values of AUC_0–24_, AUC_0–*∞*_, AUMC_0–24_, and AUMC_0–*∞*_ of VA from the optimized formulation were significantly higher than those of standard VA. Moreover, the mean residence time of VA in the systemic circulation, which was extended by about 3 times for VA from pharmacosome nanoparticles after oral administration. Based on the AUC_0–*∞*_ values, the calculated relative oral bioavailability of VA from pharmacosomes nanoparticles was about 384% when compared to standard VA.

The improvement in the bioavailability of VA from pharmacosomes nanoparticles may be due to the reduction of the size of the drug which in turn enhances its water solubility. Also, when the size of the water-soluble drug is smaller than the diameter of the pore of the biomembrane, the drug can penetrate easily. The driving force is constituted by the hydrostatic pressure or the osmotic difference across the biomembrane due to which the bulk flow of water along with small solid molecules occurs through such aqueous channels [55]. In addition, the small particle size has a longer circulation time, as it is not recognized by the RES [40], which in turn causes the drug to better reach the site of action, with enhancement in the drug’s bioavailability [55].

### In vivo pharmacodynamic study

The present study revealed a potent cardioprotective effect of VA against isoprenaline-induced myocardial injury due to its antioxidant, anti-inflammatory, and cytoprotective properties.

#### Effect of VA on serum CK-MB activity and cardiac MDA and GSH contents

Results of the current study showed a significant increase in CK-MB activity upon isoprenaline injection as an indication of the produced myocardial injury. Treatment with VA produced a marked cardioprotective effect that was reflected through the significant decrease in the CK-MB activity, while the optimized formulation normalized the CK-MB activity with no significant difference from the normal group (Table 7). Previous studies have demonstrated that the cardioprotective effect of VA may be due to its antioxidant and free radical scavenging characteristics [56].

**Table 7:** Effect of VA on serum CK-MB activity and cardiac MDA and GSH contents

	**Normal**	**MI control**	**VA**	**VA formulation**
**CK-MB (U/L)**	111.0 ± 2.3	711.0^a^ ± 58.3	466.5^ab^ ± 25.1	244.0^bc^ ± 56.8
**MDA (nmol/g)**	35.81 ± 1.2	62.04^a^ ± 3.5	47.93^ab^ ± 3.7	33.61^bc^ ± 2.9
**GSH (μmol/g)**	3.59 ± 0.3	2.15^a^ ± 0.1	3.90^ab^ ± 0.4	7.05^bc^ ± 0.3

The data is presented as mean ± SEM (*n* = 6)

^a^significant difference from normal group

^b^significant difference from MI control group

^c^significant difference from VA group (at *p* < 0.05)

There was a noticeable increased oxidative stress in the heart tissue of the MI control group, revealed by the significant increase in the cardiac MDA content accompanied by a significant suppression of GSH content. Treatment with VA resulted in a significant decrease in MDA, and a significant increase in GSH in the heart tissue. The optimized formulation normalized the myocardial MDA content and produced a marked elevation in the GSH content compared to the normal and MI control groups (Table 7). The current study demonstrated the antioxidant effect of VA through the significant decrease in lipid peroxidation, accompanied by a noticeable increase in reduced glutathione in the VA-treated group compared to the isoprenaline control rats, whereas the optimized formulation (vanillic formulation) resulted in a more powerful antioxidant effect in the heart tissue compared to the VA-treated group. These results are in agreement with previous studies that highlighted the antioxidant effects of VA [6, 57].

#### Effect of VA on myocardial expression of miR-217, MAPK, and PI3K

**Fig. 11:**
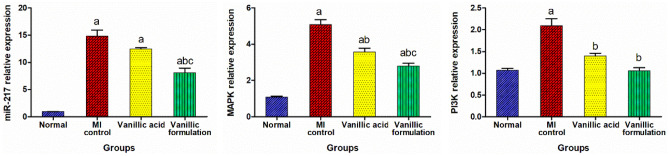
Effect of VA on expression of miR-217, MAPK and PI3K in heart tissue. The data are presented as mean ± SEM (*n* = 6). a: significant difference from normal group, b: significant difference from MI control group, c: significant difference from VA group (at *p* < 0.05)

Upon further investigation of the underlying mechanism of the cardioprotective effect of VA, results of the current study showed a significant increase in the expression level of miR-217, MAPK, and PI3K in the heart tissue upon isoprenaline injection. On the other hand, treatment with VA significantly decreased MAPK and PI3K expression, by 29.8% and 33.2%, respectively, accompanied by a non-significant change in miR-217 expression compared to the MI control group. The vanillic formulation group showed significantly suppressed expression of miR-217 and MAPK by 45.3% and 45.1%, respectively, compared to the MI control group, and successfully normalized the PI3K expression level (Fig. 11).

Previous studies have demonstrated the involvement of stimulation of mitogen-activated protein kinase (MAPK) in the pathogenesis of myocardial cell degeneration and infarction [58, 59]. The intracellular signaling pathways MAPK and PI3K/AKT/NF-κB were demonstrated to be vital in both regulating inflammatory mediators and cell structure alterations in a sequential cascade manner in many diseases including myocardial infarction [60–62]. In comparison to the isoprenaline control group, treatment with VA revealed a significant reduction in the expression levels of MAPK and PI3K, in heart tissue. On the other hand, treatment with the optimized formulation resulted in a significant suppression of the expression levels of MAPK and PI3K in heart tissue. These results suggest that the cardioprotective effect of VA can be due to the inactivation of the MAPK signaling pathway with further inhibition of the pro-inflammatory mediators. This effect of VA was previously reported in the study of [60] who suggested that VA can attenuate articular cartilage degeneration in osteoarthritis models via inhibition of the MAPK signaling pathway.

MicroRNAs are small non-coding RNAs that regulate the expression of genes inhibiting the translation process by destroying particular target mRNA. MicroRNAs were reported to play a vital role in many cardiovascular diseases’ pathogenesis, including myocardial infarction and heart failure [63]. MiR-217 was reported to have a main effect in the pathogenesis of cardiovascular diseases via modulating some pathways including the MAPK/NF-κB pathways. MiR-217 expression increases during endothelial cell damage, myocardial cell degeneration, and heart failure. In addition, it was reported that high plasma levels of miR-217 are associated with elevated cardiovascular disease risk in humans [64]. Results of the current study support these findings as it showed a significant increase in miR-217 expression in the MI control rats compared to the normal rats. On the other hand, treatment with the optimized pharmacosomal formulation resulted in a significant decrease in the myocardial expression of miR-217 compared to the isoprenaline control group.

#### Effect of VA on IL-6 and AKT

**Fig. 12:**
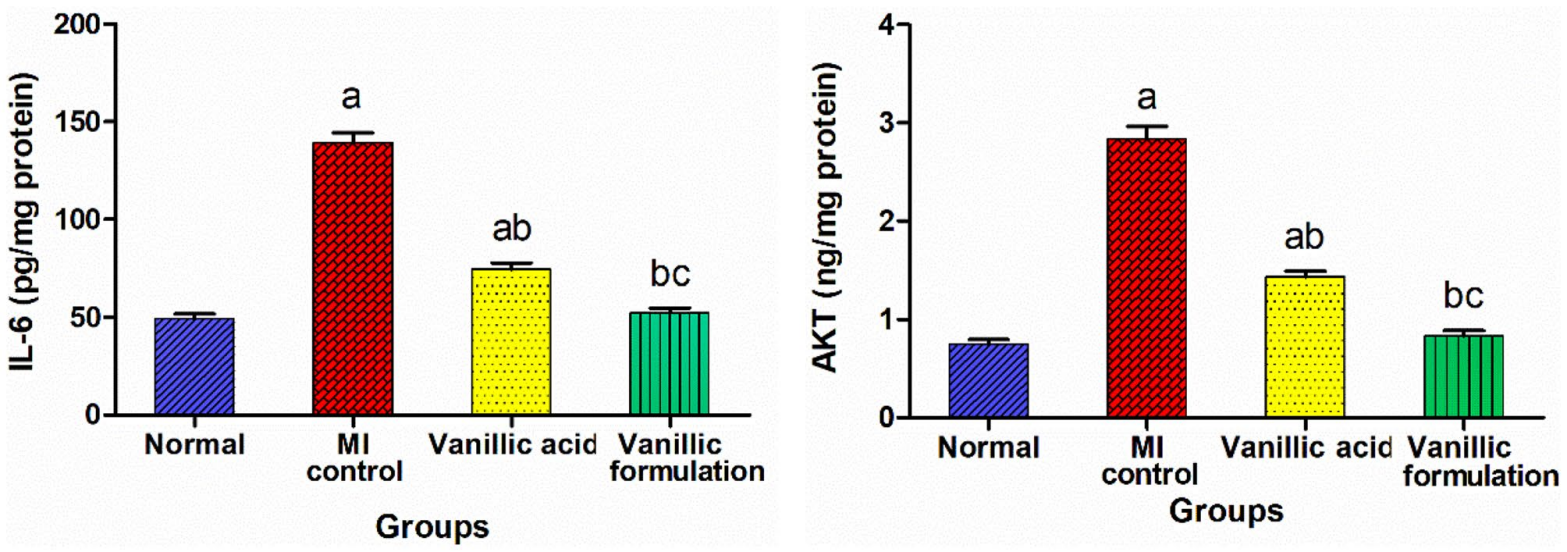
Effect of VA on IL-6 and AKT. The data is presented as mean ± SEM (*n* = 6). a: significant difference from normal group, b: significant difference from MI control group, c: significant difference from VA group (at *p* < 0.05)

A significant increase in IL-6 and AKT levels in heart tissue was observed in the MI control group compared to the normal group. Conversely, treatment with VA significantly suppressed the myocardial levels of IL-6 and AKT by 46.5% and 49.4%, respectively, compared to the MI control rats. Moreover, the rats treated with the optimized formulation exhibited significantly suppressed IL-6 and AKT levels by 62.3% and 70.7%, respectively, compared to the MI control with a non-significant difference from the normal group (Fig. 12). As stated earlier, the intracellular signaling pathways MAPK and PI3K/AKT/NF-κB affect myocardial infarction [60–62]. It was observed that VA treated group showed a significant reduction in the expression levels of AKT in heart tissue as compared to the isoprenaline control group, with a subsequent inhibition of the myocardial contents of the proinflammatory mediators IL-6. On the other hand, treatment with the optimized formulation resulted in a significant suppression of the expression levels of AKT in heart tissue, accompanied by a subsequent normalization of the myocardial contents of IL-6 with a non-significant difference from the normal rats.

#### Effect of VA on cardiac content of TNF-α

**Fig. 13:**
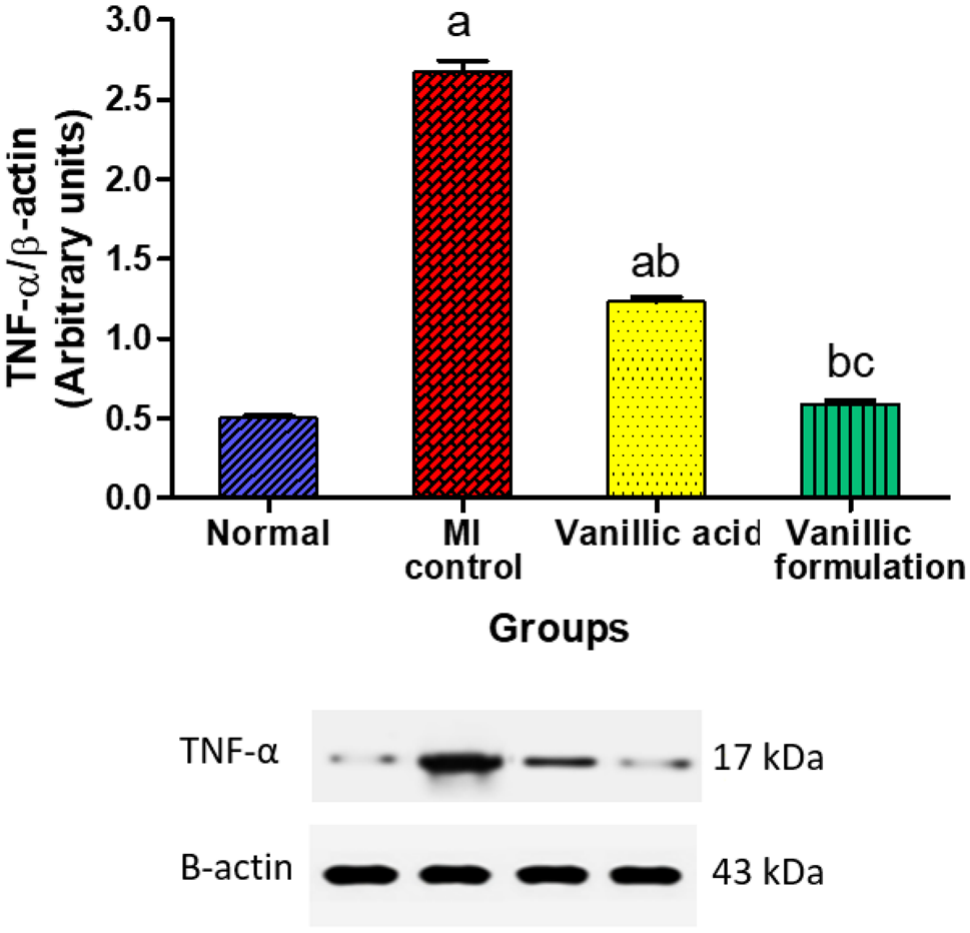
Effect of VA on cardiac content of tumor necrosis factor-α (TNF-α). The data are presented as mean ± SEM (*n* = 6). a: significant difference from normal group, b: significant difference from MI control group, c: significant difference from VA

Results of the current study revealed that the cardiac TNF-α content significantly increased by 5.4 folds in the MI control group compared to the normal rats. On the other hand, VA treatment resulted in a significant decrease in TNF-α content in heart tissue by 54.1% compared to the MI control group. The rats treated with the optimized formulation showed normal TNF-α myocardial content with a non-significant difference from the normal rats (Fig. 13).

#### Effect of VA on immunohistochemical reactivity of NF-kB

**Fig. 14:**
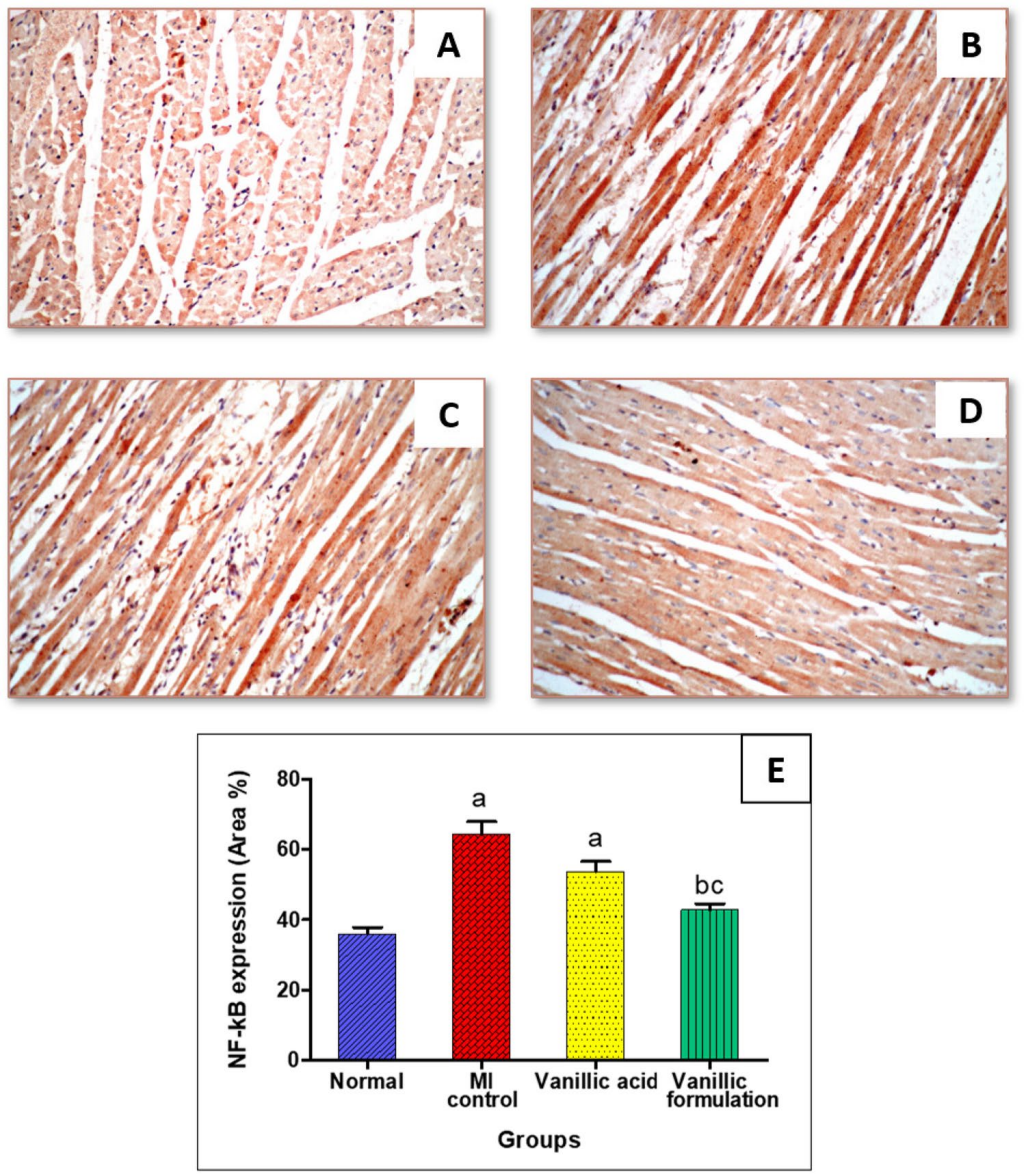
Immunostaining of nuclear factor-kB (NF-kB) in heart tissue of rats (× 40) (**A**) Normal group, (**B**) MI control group, (**C**) Vanillic acid treated group, (**D**) Vanillic formulation treated group, (**E**) represents a comparative quantification of the immunohistochemical expression for NF-kB in rats’ heart tissue from all groups. The severity of the immunoactivity is depending on the intensity and dispersion of the brown coloration computed as area % using the ImageJ software. a: represents a significant difference from the normal group, b: represents a significant difference from MI control group, c: significant difference from VA group (at *p* < 0.05)

The immuno-staining for NF-kB showed weak expression in the heart tissue of the normal rats (Fig. 14A). The expression of NF-kB noticeably increased in the heart tissue upon injection of isoprenaline HCl (Fig. 14B). VA treatment resulted in a non-significant decrease of NF-kB expression in heart tissue (Fig. 14C). On the other hand, the vanillic formulation showed marked a decrease in myocardial NF-kB expression with a significant improvement from the inducted group (Fig. 14D). Comparative quantification of the immunohistochemical expression for NF-kB in heart tissue of rats from all groups is presented in Fig. 14E.

#### Effect of VA treatment on myocardial histological structure

**Fig. 15:**
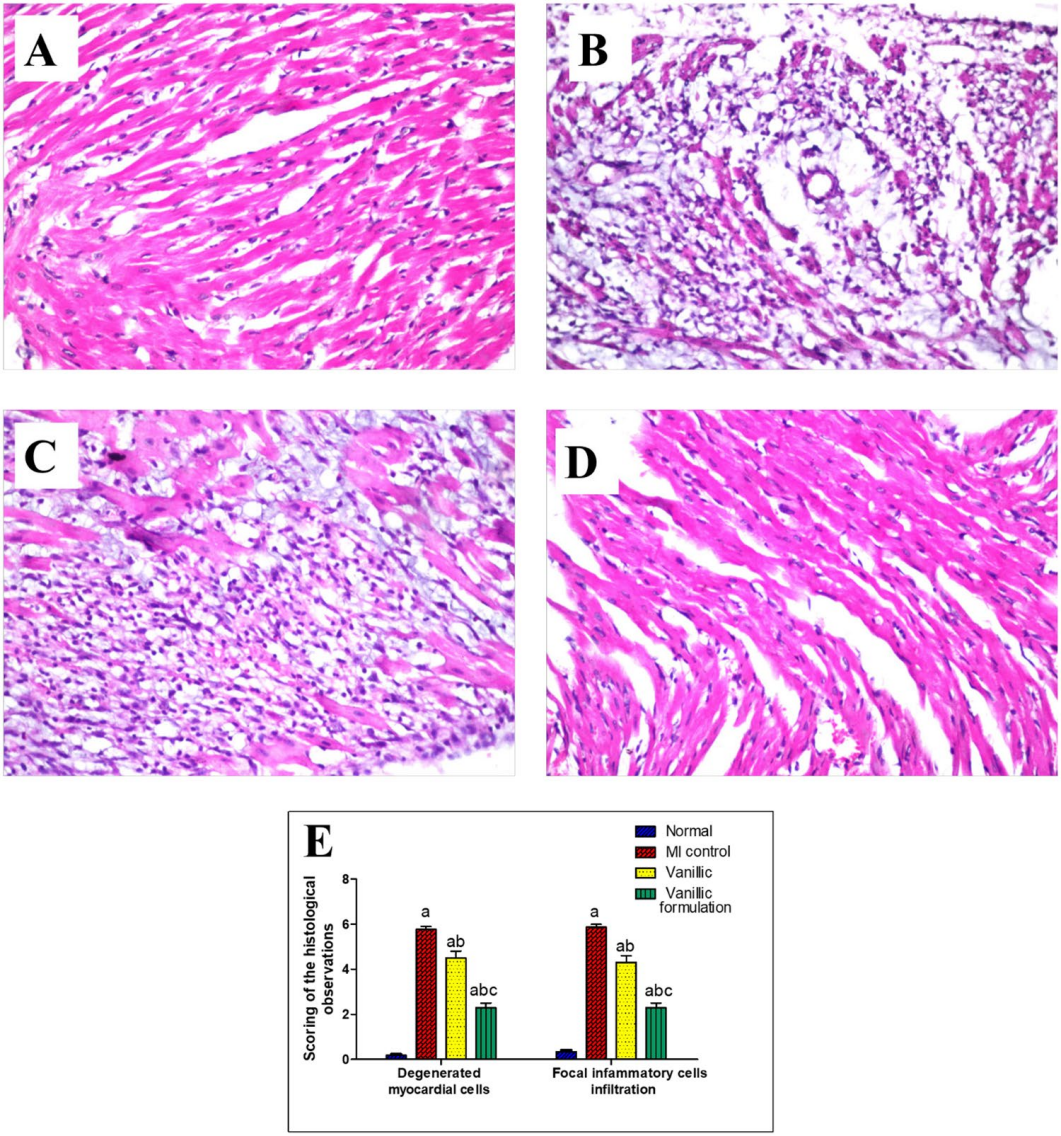
Effect of VA treatment on myocardial histological structure (H&E × 40) (**A**) Normal group, (**B**) MI control group, (**C**) Vanillic acid treated group, (**D**) Vanillic formulation treated group, (**E**) Scoring of the histological observations in myocardial tissue from all groups. a: significant difference from the normal group, b: significant difference from MI control group, c: significant difference from VA group (at *p* < 0.05)

Examination of the heart section of the rats showed the normal histological structure of the myocardial bundles with one centrally nucleated cardiomyocyte (Fig. 15A). Isoprenaline injection produced multiple areas of degenerated myocardium with inflammatory cells infiltration in a diffused manner all over the myocardial bundles (Fig. 15B). Rats pretreated with VA showed multiple areas of degeneration and inflammatory cell infiltration with minor improvement from the isoprenaline control group (Fig. 15C). On the other hand, hearts of the rats pretreated with the optimized formulation showed few focal areas of degenerative changes and inflammatory infiltration, and most of the myocardium showed intact histological structure (Fig. 15D). Scoring of the histological observations in the myocardium is presented in Fig. 14E.

The optimized formulation surprisingly normalized the histological structure of the myocardial tissue with significant improvement from the group treated with VA alone. This probably could be due to the antioxidant and free radical scavenging characteristics of VA [56], which were greatly enhanced when formulated into nanoparticles.

As can be observed, the formulation of VA into pharmacosomes nanoparticles not only enhanced VA bioavailability but also enhanced its cardioprotective effect. This is probably due to its enhanced solubility, which in turn increases its therapeutic activity.

#### Effect of VA on ECG monitoring

**Fig. 16:**
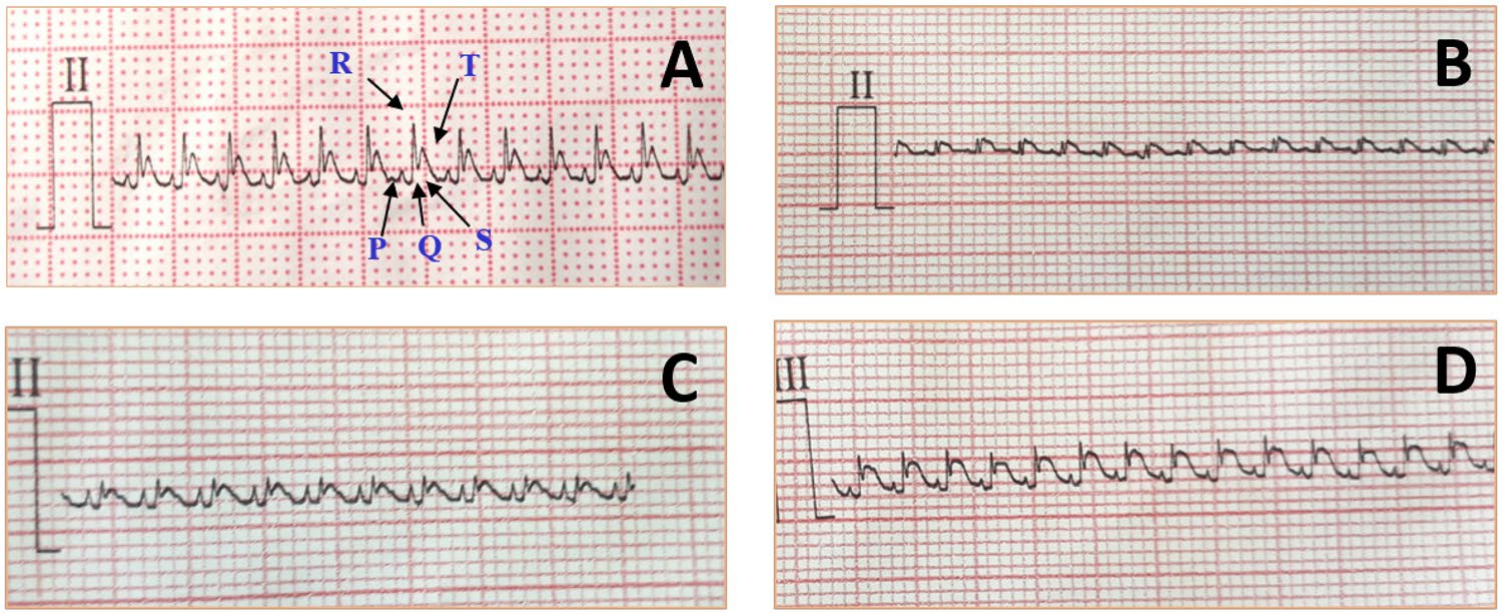
Effect of VA treatment on ECG (lead II) of rats. (**A**) ECG chart of a normal rat; (**B**) ECG chart of rats from MI control group; (**C**) rats pretreated with vanillic acid; (sD) rats pretreated with the vanillic acid formulation

A regular ECG pattern with distinct P, QRS, and T waves was visible on the lead II ECG trace of a healthy rat. Because the electromotive forces in the infarcted areas have been lost, the R wave amplitude in the ECG analysis of the isoprenaline-treated group has significantly decreased when compared to that of the normal group. Compared to the MI control group, pretreatment with vanillic acid revealed a slight elevation in the R wave. On the other hand, rats pretreated with the vanillic formulation had a significantly higher R wave amplitude (Fig. 16). Results of the ECG monitoring revealed the potential effect of the optimized formula on the heart.

## Conclusion

In the present study, VA-loaded pharmacosomes were developed by applying a central composite design, where the effect of each PC:VA molar ratio and the precursor concentration were studied. Numerical optimization of the particle size, polydispersity index, and zeta potential was achieved based on a desirability approach. An optimized formulation was successfully prepared and showed good stability with a sustained release profile of the drug up to 48 h. An improvement in the bioavailability of the optimized formulation was achieved, which was almost two times better than standard VA. Furthermore, VA had a promising cardioprotective effect, when tested in vivo via inhibition of the MAPK pathway, with subsequent inhibition of PI3k/NF-κB signaling, in addition to its antioxidant effect. However, the optimized formulation was more potent than standard VA and resulted in the normalization of many of the oxidative stress and inflammatory biomarkers. Thus, pharmacosomes successfully enhanced the bioavailability of VA and showed a promising cardioprotective activity when formulated with VA.

## Data Availability

Raw data were generated at MSA University. Derived data supporting the findings of this study are available from the corresponding author M.H.S. Dawoud on request

## Declarations

### Animal studies

All institutional and national guidelines for the care and use of laboratory animals were followed.
